# *E. coli* primase and DNA polymerase III holoenzyme are able to bind concurrently to a primed template during DNA replication

**DOI:** 10.1038/s41598-019-51031-0

**Published:** 2019-10-08

**Authors:** Andrea Bogutzki, Natalie Naue, Lidia Litz, Andreas Pich, Ute Curth

**Affiliations:** 1 Institute for Biophysical Chemistry, Hannover Medical School, Hannover, 30625 Carl-Neuberg-Str. 1, Germany; 2 Institute for Toxicology, Hannover Medical School, Hannover, 30625 Carl-Neuberg-Str. 1, Germany; 3 Present Address: Inamed GmbH, Gauting, 82131 Germany

**Keywords:** DNA synthesis, Biophysical chemistry

## Abstract

During DNA replication in *E. coli*, a switch between DnaG primase and DNA polymerase III holoenzyme (pol III) activities has to occur every time when the synthesis of a new Okazaki fragment starts. As both primase and the χ subunit of pol III interact with the highly conserved C-terminus of single-stranded DNA-binding protein (SSB), it had been proposed that the binding of both proteins to SSB is mutually exclusive. Using a replication system containing the origin of replication of the single-stranded DNA phage G4 (G4ori) saturated with SSB, we tested whether DnaG and pol III can bind concurrently to the primed template. We found that the addition of pol III does not lead to a displacement of primase, but to the formation of higher complexes. Even pol III-mediated primer elongation by one or several DNA nucleotides does not result in the dissociation of DnaG. About 10 nucleotides have to be added in order to displace one of the two primase molecules bound to SSB-saturated G4ori. The concurrent binding of primase and pol III is highly plausible, since even the SSB tetramer situated directly next to the 3′-terminus of the primer provides four C-termini for protein-protein interactions.

## Introduction

The exact replication of the genome is a prerequisite for the division of the bacterial cell. Several proteins and protein complexes have to cooperate at the bacterial replication fork in order to fulfil this demanding task. In *E. coli*, the hexameric helicase (DnaB), primase (DnaG), single-stranded DNA-binding protein (SSB) and DNA polymerase III holoenzyme (pol III) as the replicative DNA polymerase are necessary for the progression of DNA synthesis. Pol III is composed of three sub-complexes: 1. the αεθ core that comprises the 5′−3′ polymerase and the 3′−5′ proofreading activities, 2. the β_2_ sliding clamp that confers processivity to the core and 3. the (τ/γ)_3_δδ′ψχ clamp loader. The clamp loader not only loads the ring-shaped β_2_ clamp onto the DNA, but also organises the whole replication fork, undergoing protein-protein interactions with the core, the helicase and SSB^1–3^. Depending on the number of τ proteins incorporated in the clamp loader complex, up to three cores can be contained in the pol III holoenzyme. At least two cores are required for the simultaneous replication of the two parental DNA strands^4^. The tetrameric SSB protein not only protects the vulnerable single-stranded DNA and holds it in a proper conformation, but also engages in several interactions with proteins involved in DNA replication, repair and recombination via its highly conserved amphipathic C-terminus^5^. Amongst these proteins are the χ subunit of the clamp loader^6–11^ and DnaG primase^12,13^. Since pol III is not able to synthesize DNA *de novo*, each newly synthesized DNA strand starts with an RNA primer produced by primase. Primase is composed of three domains: 1. an N-terminal zinc-binding domain that is responsible for the recognition of the template, 2. a central RNA-polymerase domain and 3. a C-terminal helicase-binding domain^14^. The helicase-binding domain also interacts with the amphipathic C-terminus of SSB via a hydrophobic pocket surrounded by basic residues^12^.

As the *E. coli* genome consists of double-stranded, antiparallel DNA, and pol III synthesizes DNA exclusively in 5′−3′ direction, only the leading strand can be synthesized continuously, whereas the lagging strand has to be synthesized in fragments of about 1000 to 2000 nucleotides, the so-called Okazaki fragments^1–3^. Since each Okazaki fragment starts with an RNA primer that is synthesized by primase and subsequently elongated by pol III, a primase-to-polymerase switch has to occur during the synthesis of each Okazaki fragment. It has been shown that both the χ subunit of the clamp loader and DnaG primase interact with the amphipathic C-terminus of SSB^9,10,12,13^. A model has been proposed in which DnaG primase is displaced from the C-terminus of SSB by the χ subunit of the clamp loader in order to allow for the loading of the β_2_ sliding clamp^13^; it assumes that χ and primase cannot bind to SSB at the same time. However, this model has been controversially discussed, as SSB forms stable tetramers and four C-termini are available at the SSB protein bound next to the primer. This leads to the question why χ should interact preferentially with the C-terminus of the one SSB protomer bound to primase^2^. It has also been speculated that a gain-of-function defect in the temperature-sensitive SSB-113 mutant used to develop the model of primase displacement by χ caused the observed effect^3^. Therefore, we wanted to test whether primase and pol III are able to bind concurrently to a primed template or whether primase has to be displaced to allow for elongation of the primer by pol III.

**Figure 1:**
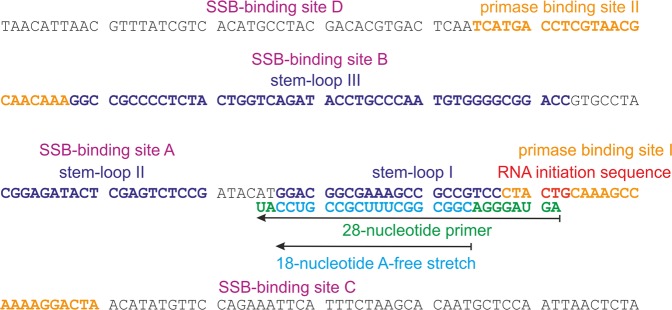
Sequence of the origin of replication of single-stranded DNA phage G4 (G4ori). Secondary structure elements, RNA initiation sequence and binding sites of primase and SSB are indicated^17,18^. The 28-nucleotide full-length primer that can be synthesized from G4ori by DnaG primase contains an 18-nucleotide stretch free of adenine nucleotides. This stretch starts at position 9 with the first cytosine that is incorporated into the primer.

DnaG primase on its own synthesizes primers very inefficiently^15^. It needs other factors such as SSB, DnaB helicase and other components of the primosome in order to start primer synthesis. At the origin of replication of the *E. coli* chromosome, primer synthesis requires the presence of the initiator proteins DnaA, DnaB and the helicase loader DnaC^2^. The origin of the single-stranded DNA phage G4 (G4ori), which is composed of a complex secondary structure, however, allows primer synthesis in the presence of primase and SSB only^16^. Primer synthesis by primase in the presence of SSB is initiated at the thymine residue of the trinucleotide ^5′^CTG^3′^ located 3′ of the three stem-loop structures of G4ori^17^ (Fig. 1). When SSB binds to G4ori, SSB-free regions of approximately 30 nucleotides are created on both sides of the stem-loop structure, allowing primase to bind to ssDNA and SSB simultaneously^18^. In the presence of ribonucleotides (rNTPs), primase is able to synthesize a primer of up to 28 nucleotides and the addition of pol III and deoxyribonucleotides (dNTPs) will result in the conversion of the whole phage genome into double-stranded DNA^19,20^.

To ensure that our investigations were comparable to the study proposing the mutual exclusive binding of primase and χ to SSB^13^, we used single-stranded M13Gori DNA (ssM13Gori) as a template. In this template, G4ori is integrated into M13mp8 DNA^17^. In sedimentation velocity experiments in an analytical ultracentrifuge, free proteins were resolved from those in complex with ssM13Gori and we found that primase and DNA pol III bind concurrently to the primed template. Even pol III-mediated primer elongation by several nucleotides did not lead to a significant primase displacement. Elongation by more than 10 nucleotides, however, led to the dissociation of one primase molecule, whereas complete conversion of ssM13Gori to its double-stranded form resulted in complete release of primase.

## Results

### Specific binding of DnaG primase to G4ori

**Figure 2:**
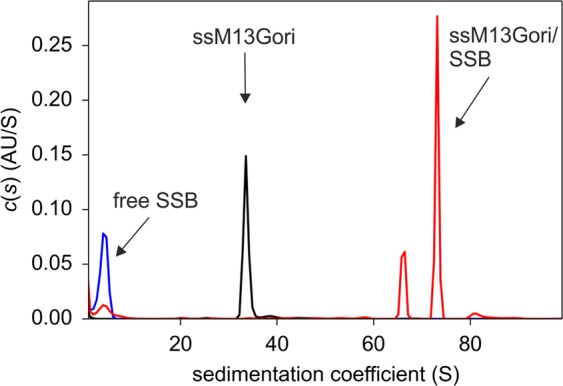
Binding of SSB to ssM13Gori. Sedimentation velocity analysis with absorbance detection at 280 nm was used to analyse 20 nM ssM13Gori (black), 5 µM SSB (blue) and a mixture thereof (red). The two peaks at about 70 S represent complexes of linear and circular ssM13Gori with SSB (details see text). Experiments were performed in 20 mM Tris/HCl, 150 mM NaCl, 10 mM MgCl_2_, 1 mM TCEP, pH 7.5 at 20 °C.

We first analysed our ssM13Gori-preparation by sedimentation velocity AUC experiments using absorbance detection. The experiments revealed a single peak with a sedimentation coefficient of about 34 S; addition of a slight excess of SSB resulted in the formation of two complexes, sedimenting at about 70 S (Fig. 2). A similar behaviour was observed with ssM13mp8 as a control (data not shown). Most probably the major, faster sedimenting peak represents a complex of SSB with circular ssM13Gori, whereas the slower one represents an SSB complex with the linear form, potentially originating from single-strand breaks that occurred during ssDNA purification. On native agarose gels, the prepared ssM13Gori migrated as a single band (data not shown). When alkaline loading buffer was used, however, two bands were observed, with the one migrating more slowly corresponding to about three-quarters of the total signal (Supplementary Fig. S2). To test whether the two bands really represented circular and linear forms of ssM13Gori, we annealed a complementary oligonucleotide to the ssDNA to generate a PvuII recognition side. After cleavage with PvuII, the DNA migrated as a single band with the same velocity as the faster migrating band before cleavage (Supplementary Fig. S2), confirming that the main band was circular ssM13Gori DNA.

**Figure 3:**
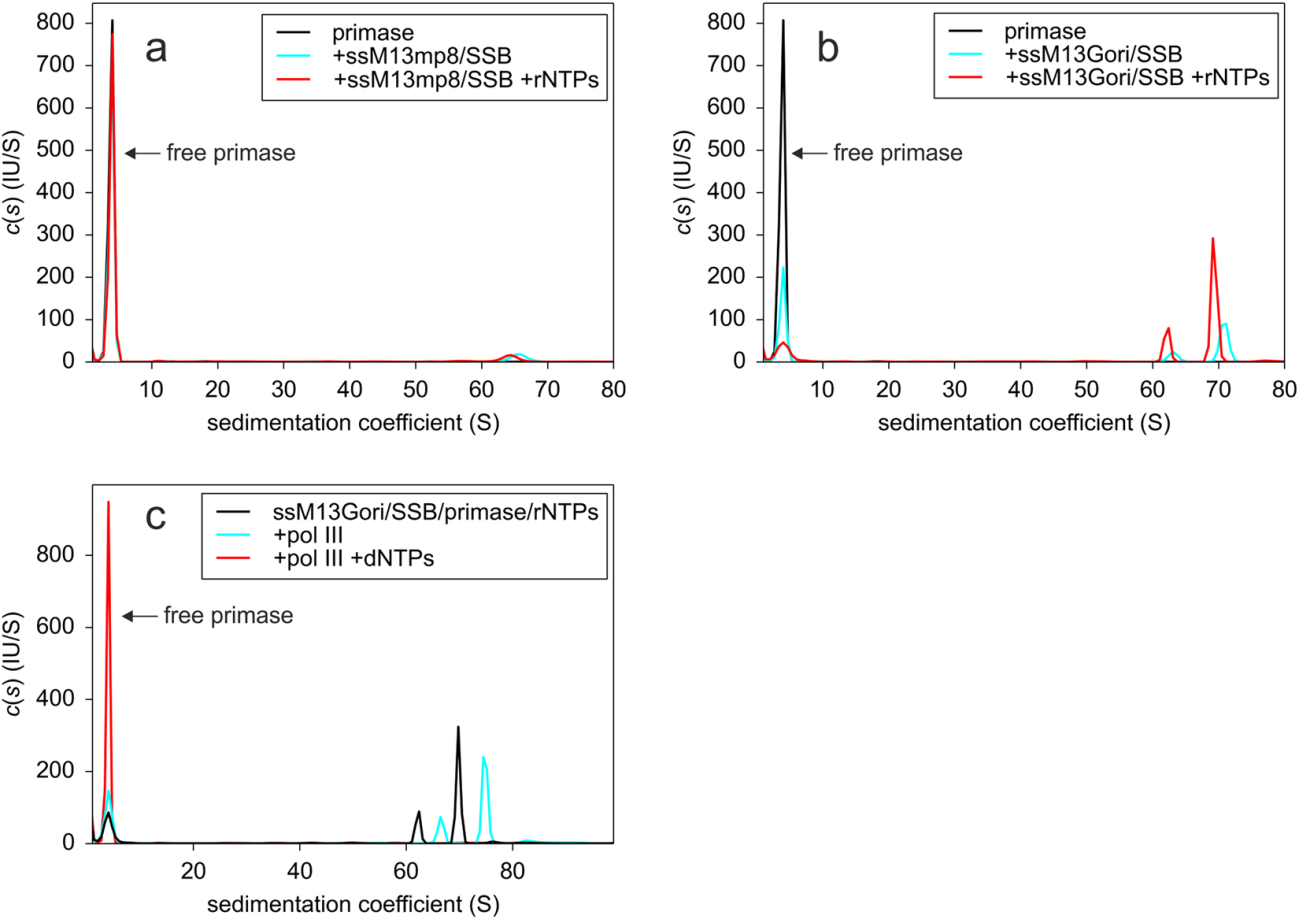
Interaction of primase and pol III with ssM13Gori/SSB complexes. (**a**) Analysis of the interaction of 10 nM DL-DnaG primase (black) with 6 nM ssM13mp8/SSB complexes in the absence (cyan) and presence (red) of 2 mM ATP and 0.1 mM each of UTP, GTP and CTP by AUC using fluorescence detection. Independent of the presence of rNTPs, only a very small amount of primase binds to the ssDNA/SSB complex. (**b**) As in panel A but 5 nM ssM13Gori/SSB complex was used as a template. Primase binds specifically to G4ori; in the presence of rNTPs the amount of bound primase increases significantly. (**c**) 5 nM ssM13Gori/SSB/primase complexes in the presence of rNTPs (black, corresponding to the red curve in panel b) were incubated with 15 nM pol III in the absence (cyan) and presence (red) of 0.1 mM dNTPs. In the presence of pol III alone, hardly any primase was displaced and the peaks of the linear and circular forms of the ssM13Gori/SSB/primase complexes shifted from 62 S and 70 S to 67 S and 75 S, respectively. This reveals that pol III enters the complex without displacing primase. The addition of dNTPs, which results in DNA synthesis, however, leads to a complete displacement of primase from DNA. Experiments were performed in 20 mM Tris/HCl, 150 mM NaCl, 10 mM MgCl_2_, 1 mM TCEP, pH 7.5 at 20 °C.

**Figure 4:**
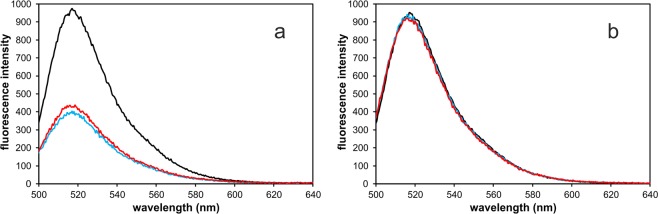
Fluorescence of DL-DnaG primase decreases by binding to G4ori. (**a**) Emission spectra of DL-DnaG (18 nM) before (black) and after addition of a 3-fold molar excess of ssM13Gori/SSB in the absence (cyan) and presence (red) of 2 mM ATP and 0.1 mM each of UTP, GTP and CTP. Measured fluorescence intensities were corrected for dilution. In the presence of ssM13Gori, the fluorescence signal of DL-DnaG dropped by more than a factor of 2. (**b**) When the same experiment as shown in panel A was performed with ssM13mp8/SSB, no change in fluorescence was observed. Experiments were performed in 20 mM Tris/HCl, 150 mM NaCl, 10 mM MgCl_2_, 1 mM TCEP, pH 7.5 at 20 °C.

ssM13Gori comprises about 8600 nucleotides and more than 100 SSB tetramers are expected to be bound in the ssM13Gori/SSB complex. In order to facilitate the detection of a single primase molecule binding to these huge complexes, we fluorescently labelled DnaG with DyLight488 (DL-DnaG) and used AUC with fluorescence detection. Since all other components were unlabelled, only the primase signal was detected. Free DL-DnaG showed a sedimentation coefficient of 3.8 S (Fig. 3a,b); this value is virtually identical to the s-value of unlabelled, monomeric primase measured by AUC using absorbance detection (data not shown). Addition of naked ssM13Gori or ssM13mp8 to DL-DnaG did not result in complex formation (data not shown). This is consistent with previous findings that SSB is necessary for a stable interaction of primase with G4ori^13,16,18^. Whereas only a very small amount of primase bound to an ssM13mp8/SSB complex (Fig. 3a), most probably by an exclusive interaction with SSB, the presence of G4ori in the ssM13Gori/SSB complex resulted in the binding of a significant amount of DL-DnaG due to the specific interaction of DL-DnaG with SSB-saturated G4ori (Fig. 3b). This specific binding caused a noticeable decrease in the total fluorescence intensity of DL-DnaG. Accordingly, emission spectra of DL-DnaG, measured in a static fluorometer, showed a drop in fluorescence intensity by more than a factor of two when an excess of ssM13Gori/SSB complex was added, either in the absence or presence of rNTPs (Fig. 4a). No change in fluorescence was observed after addition of ssM13mp8/SSB complex (Fig. 4b), indicating that the quantum yield of DL-DnaG is significantly decreased by binding specifically to G4ori/SSB. Since the quantum yield of free DL-DnaG self-evidently does not depend on the presence of ssM13Gori/SSB, the decrease in fluorescence of free DL-DnaG can be used to infer the amount of bound DL-DnaG in the AUC experiments.

**Figure 5:**
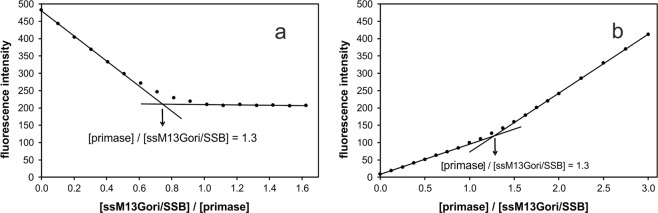
One primase binds to G4ori in the absence of rNTPs. In the absence of rNTPs (**a**) 10 nM DL-DnaG were titrated with ssM13Gori/SSB or (**b**) 5 nM ssM13Gori/SSB were titrated with DL-DnaG. Measured fluorescence intensities were corrected for dilution. In both titrations, the affinity was too high to be determined and a stoichiometry of 1.3 primases per ssM13Gori/SSB was obtained from the point of intersection of the tangents. Experiments were performed in 20 mM Tris/HCl, 150 mM NaCl, 10 mM MgCl_2_, 1 mM TCEP, pH 7.5 at 20 °C.

As the quantum yield of DL-DnaG changes when the protein binds specifically to ssM13Gori/SSB complexes, this interaction can be characterised by titrations in a fluorometer. When titrating ssM13Gori/SSB complexes to 10 nM DL-DnaG in the absence of rNTPs, stoichiometric titration curves were observed (Fig. 5a). From the equivalence point, a stoichiometry of 1.3 primases per ssM13Gori/SSB complex was determined. The binding affinity of primase, however, is so high that it cannot be determined from titrations using concentrations in the lower nanomolar range. Therefore, it is assured that virtually all G4ori is bound by primase in our AUC experiments, since we always used an excess of primase. Inverse titrations, where DL-DnaG was added to ssM13Gori/SSB, yielded the same stoichiometry (Fig. 5b).

Thus, one primase molecule binds to G4ori in the absence of rNTPs. In AUC experiments, addition of rNTPs to a mixture of DL-DnaG and ssM13Gori/SSB resulted in a distinct reduction of free primase, to the effect that it got bound to ssM13Gori/SSB almost entirely (Fig. 3b). Accordingly, the fluorescence intensity of the DNA-containing complexes increased considerably. Since we used a 2-fold excess of DL-DnaG over DNA, it is highly probable that a second primase molecule bound to G4ori in presence of rNTPs. The slight decrease in s-value after addition of rNTPs most likely indicates a conformational change of the complex due to the binding of the second primase molecule. Determination of the exact binding stoichiometry in the presence of rNTPs by fluorescence titrations was precluded by the long equilibration times needed after each addition of SSB-saturated DNA or DL-DnaG, respectively. The necessity of binding two primase molecules for effective primer synthesis has already been described previously^16,18,21^.

### DNA polymerase III holoenzyme does not displace primase from ssM13Gori

Earlier experiments using an ssM13Gori replication system led to the development of a model proposing that by competing for the binding to the C-terminus of SSB, primase is displaced by the χ subunit of the pol III clamp loader^13^. However, SSB forms stable tetramers and each of those provides four C-termini for protein-protein interactions. Consequently, even the SSB situated directly next to the 3′-terminus of the primer, where the clamp loader is expected to bind, should still have three binding sites available. This raises the question whether primase and DNA polymerase can concurrently bind to the template/primer via SSB.

To address this question, we added a 1.5-fold molar excess of pol III over primase to a complex of DL-DnaG and ssM13Gori/SSB and free and bound primase were resolved by AUC. The experiment was performed in the presence of rNTPs to allow for primer formation, but in the absence of dNTPs to prevent DNA synthesis. Whereas the signal of free DL-DnaG hardly changed upon addition of pol III, indicating that primase stayed bound to the ssDNA/SSB complex, the peaks of the ssM13Gori/SSB/DL-DnaG complexes shifted to significantly higher s-values (Fig. 3c, cyan). This increase in the sedimentation coefficients can only be explained by the binding of pol III to the primed complex. Since DL-DnaG produces the signal detected, pol III and primase must be bound concurrently to the same protein-DNA complex. Even a 10-fold excess of pol III holoenzyme over DL-DnaG did not lead to a significant displacement of DL-DnaG from G4ori/SSB (data not shown).

**Figure 6:**
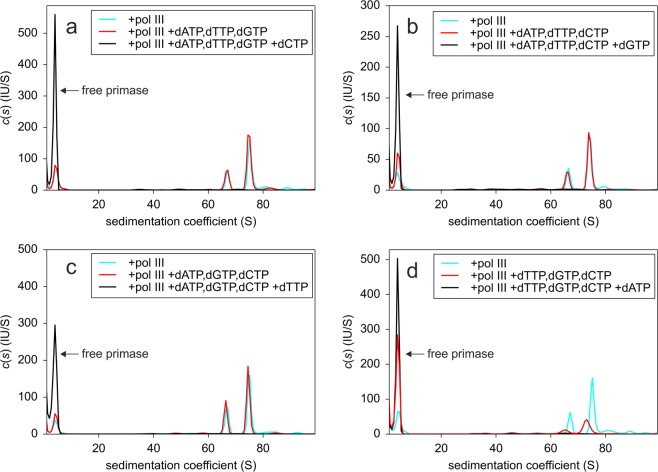
Effect of the addition of different mixtures of three dNTPs on the displacement of primase from ssM13Gori/SSB by pol III. A complex of 5 nM ssM13Gori/SSB/DL-DnaG in the presence of 2 mM ATP and 0.1 mM each of UTP, GTP and CTP was incubated with 15 nM pol III in the absence (cyan) and presence (red) of 0.1 mM each of a mixture of (**a**) dATP, dTTP, dGTP (**b**) dATP, dTTP, dCTP (**c**) dATP, dGTP, dCTP or (**d**) dTTP, dGTP, dCTP. As a control, 0.1 mM of the respective missing fourth dNTP was added to the mixtures (black). The only mixture of three dNTPs that resulted in a significant release of primase from the ssDNA/SSB complex in the presence of pol III was the one containing dTTP, dGTP and dCTP. Further addition of the respective fourth dNTP resulted in a complete displacement of primase in all cases, showing that the system was fully functional. Experiments were performed in 20 mM Tris/HCl, 150 mM NaCl, 10 mM MgCl_2_, 1 mM TCEP, pH 7.5 at 20 °C.

To check whether the system is fully functional and whether primase dissociates after complete conversion of ssM13Gori to double-stranded DNA, we conducted the same experiment in the presence of dNTPs. Under these conditions, DL-DnaG was completely displaced from the DNA (Fig. 3c, red), indicating that the system is fully functional and that conversion of ssM13Gori to its double-stranded form leads to complete primase displacement. Agarose gel analysis of samples after AUC confirmed that the presence of dNTPs resulted in DNA synthesis (Supplementary Fig. S3). Whereas binding of pol III does not result in a significant release of primase, conversion of ssM13G4ori to its double-stranded form, which also leads to dissociation of SSB, causes a complete release of primase. We then wanted to check whether the incorporation of a few nucleotides by pol III already suffices to displace primase from the complex. Therefore, we added only those three dNTPs that should be incorporated first at the 3′-end of the 28-nucleotide full-length primer (Fig. 1) observed in the G4ori system^19^. The addition of dATP, dTTP and dGTP had no impact on the amount of bound primase; if, however, dCTP was also included as a control, complete primase displacement was observed as expected (Fig. 6a). Since synthesis by primase is rather slow^15^, we could not be sure whether the full-length primer was produced to a considerable extend in our system. Therefore, we performed the experiment also with the three remaining combinations of mixtures of three dNTPs (Fig. 6b–d). To our surprise, a significant release of primase was only observed in one case, namely when a mixture of dTTP, dGTP and dCTP was provided (Fig. 6d). As all combinations of two of these nucleotides were present in one of the other mixtures as well, a primer extension by pol III by at least two nucleotides is not sufficient to displace primase.

**Figure 7:**
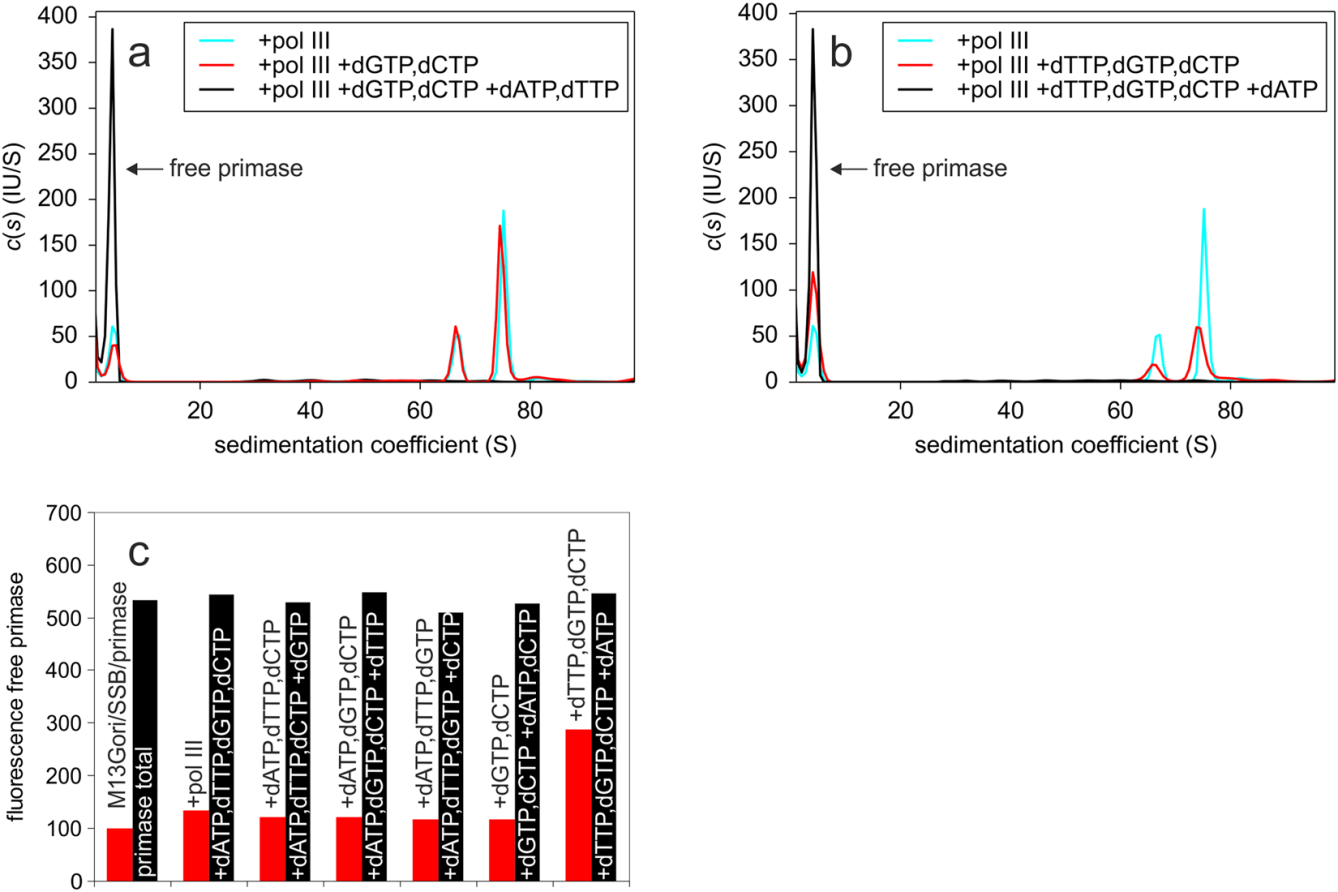
After primer synthesis in the absence of CTP, half of the primase is displaced from ssM13Gori/SSB by an addition of pol III and dTTP, dGTP and dCTP. A complex of 5 nM ssM13Gori/SSB/DL-DnaG in the presence of 2 mM ATP and 0.1 mM each of UTP and GTP was incubated with 15 nM pol III in the absence (cyan) and presence (red) of 0.1 mM each of a mixture of (**a**) dGTP, dCTP or (**b**) dTTP, dGTP, dCTP. As a control, 0.1 mM of the respective missing dNTPs was added to the mixtures (black). Whereas the addition of a mixture of dGTP and dCTP had no effect, the addition of dTTP, dGTP and dCTP resulted in a release of about half of the primase from the ssDNA/SSB complex in the presence of pol III. (**c**) Fluorescence intensities of free DL-DnaG were determined by integration of the peaks at 3.8 S in the c(s) distributions of the samples shown in panels a and b and Supplementary Fig. S4a–c. ‘Primase total’ represents the signal of free DL-DnaG in the presence of 2 mM ATP and 0.1 mM each of UTP and GTP, ‘ssM13Gori/SSB/primase’ the free DL-DnaG signal after an addition of ssM13Gori/SSB and ‘+ pol III’ the signal in presence of ssM13Gori/SSB and pol III. In the further columns, the signal of free DL-DnaG is given after having added the indicated mixtures of dNTPs in the presence of ssM13Gori/SSB and pol III. Experiments were performed in 20 mM Tris/HCl, 150 mM NaCl, 10 mM MgCl_2_, 1 mM TCEP, pH 7.5 at 20 °C.

When examining the sequence of the primer synthesized at G4ori^19^, a 18-nucleotide stretch devoid of any adenine nucleotides is conspicuous (Fig. 1). Most probably the primers synthesized in our system end in this part of the sequence. Since the first cytosine is inserted in the primer at position 9 (Fig. 1), we preincubated the complex of primase and ssM13Gori/SSB with ATP, UTP and GTP only, in order to receive a primer of defined length. Addition of dTTP, dGTP and dCTP in the presence of pol III resulted in a significant displacement of primase (Fig. 7b,c). Addition of dGTP and dCTP only, however, which should result in a primer extension by as much as seven nucleotides, showed no primase release (Fig. 7a). We also tested the remaining mixtures of three dNTPs and found no significant primase release (Supplementary Fig. S4a–c). After AUC analysis, all samples containing pol III and dNTPs were applied to agarose gel electrophoresis to check for DNA synthesis (Supplementary Fig. S5 and S6). A significant change in the running behaviour of the DNA could only be observed when all four dNTPs were present, indicating that primase displacement in the presence of dTTP, dGTP and dCTP was not due to an unexpected complete conversion of ssM13Gori to double-stranded DNA and proving that all components necessary for DNA synthesis were present in all samples.

## Discussion

Analysis of the binding of primase to ssM13Gori/SSB by fluorescence titrations revealed that in the absence of rNTPs one primase molecule binds specifically to G4ori. Sedimentation velocity experiments showed that the additional presence of rNTPs increases the amount of bound primase significantly, most probably leading to two primases bound per SSB-saturated G4ori. Scientific literature presents divergent results concerning the number of primases bound to G4ori/SSB. Whereas several groups found that the binding of two primases is necessary for efficient primer synthesis^16,18,21^, detection of radioactively-labelled primase bound to ssM13Gori/SSB after separation by size exclusion chromatography (SEC) revealed only one primase bound per G4ori^13^. In the latter case, however, only the incubation of primase with ssM13Gori/SSB was performed in the presence of rNTPs, the subsequent SEC analysis was done in their absence. The binding of the second primase in the former reports was also observed in the absence of rNTPs, but at much higher primase concentrations than used by us and by Yuzhakov *et al*.^13^ Most probably the binding of the second primase in the absence of rNTPs is weak and therefore, unless very high primase concentrations are used, it dissociates when rNTPs are removed from the reaction mixture such as by SEC separation. The binding of two molecules of primase is highly probable, since there is evidence that an interaction of the zinc-binding domain of one primase molecule and the RNA polymerase domain of another primase *in trans* is generally required for primer synthesis^22,23^. Since the DnaB helicase hexamer, which is required for the replication of the *E. coli* chromosome and stimulates primase activity, is able to bind three primase molecules, it has been speculated that the need for the interaction of two primase domains *in trans* may prevent primer synthesis in the absence of helicase^22^. In the G4ori system, where no helicase is present to optimally position the two primase molecules required for primer synthesis, three defined stem-loop structures generate two 30 nt SSB-free regions that allow the binding of two primases^18^ in an optimal distance for the interaction of their zinc-binding and RNA polymerase domains.

When we added pol III to a complex of primase and ssM13Gori/SSB in the absence of dNTPs, we found that primase and pol III can bind concurrently to SSB-saturated G4ori. Although we used a 1.5-fold excess of pol III, hardly any primase was displaced and the clear increase in sedimentation coefficient of the complex proves that polymerase enters into the complex of ssM13Gori/SSB and fluorescently-labelled primase (Fig. 3c, cyan). Therefore, our data do not support the proposed model in which a competition of pol III and primase for the binding to the C-terminus of SSB leads to the displacement of primase from the ssM13Gori/SSB complex^13^. Since SSB forms tetramers, there are four C-termini available for protein-protein interactions, even at the SSB-protein bound next to the 3′-end of the primer, allowing a concurrent binding of both primase and pol III to G4ori via SSB. When ssM13Gori is converted to its double-stranded form by pol III in presence of all four dNTPs, complete dissociation of primase is observed (Fig. 3c, red). As the stem loop structure essential for primase binding disappears during this process, and double-stranded M13Gori is not able to bind SSB, the dissociation of primase is the direct consequence. The earlier observation showing that primase can be displaced by pol III in the absence of DNA synthesis in the same ssM13G4ori system^13^ that we used now might have been caused by the analysis of the complexes in the absence of rNTPs. Similar to our experiments performed in the absence of rNTPs, the authors find only one primase molecule bound per G4ori. The affinity of a single primase to G4ori might be too weak to prevent a displacement by pol III.

To check whether a pol III-mediated extension of the primer by a few nucleotides is sufficient to cause primase dissociation, we tested the effect of all four triple combinations of dNTPs. Only the mixture of dTTP, dGTP and dCTP caused significant primase dissociation: about half of the enzyme was released from the complex (Fig. 6d, red). Since there is only one long T-free stretch in the G4ori sequence and this stretch is situated in stem-loop I (Fig. 1), the full-length 28-nucleotide primer cannot be the main product in our system. This is consistent with previous results showing that in the G4ori system 12- to 26-nucleotide-long primers are synthesized at non-optimal temperatures^15^. To obtain a primer of defined length, whose extension by pol III can be manipulated by the absence of certain dNTPs, we omitted CTP during primer synthesis. This should prevent the synthesis of primers longer than 8 nucleotides. As the rate-determining step of primer synthesis is the formation of the first phosphodiester bond or a step preceding it, no primers shorter than 8–12 nucleotides are usually observed^15,16^. Due to its low accuracy, primase is able to incorporate wrong nucleotides in the absence of CTP, thereby reading through the first guanine in the G4ori template and stopping three bases upstream at the second guanine, which may lead to primers of up to 12 nucleotides in length^18^. When checking the length of the primers synthesized by DnaG or DL-DnaG in the absence of CTP on a denaturing PAGE, we accordingly found primers of 8–12 nucleotides in length (Supplementary Fig. S7). The formation of shorter primers, however, can be enforced by the presence of dNTPs in addition to rNTPs in the primase reaction; when doing so, it turned out that the 28-nucleotide primer is not essential for effective priming of DNA synthesis by pol III, even a dinucleotide is sufficient^24^. When the primer was synthesized in our system in the absence of CTP, the addition of pol III and a mixture of dCTP and dGTP did not result in a significant primase displacement. However, about half of the primase was displaced when DNA synthesis took place in the presence of dCTP, dGTP and dTTP (Fig. 7b,c). Whereas in the first case the primer can be elongated by up to 7 nucleotides, in the latter case up to 18 nucleotides can be added. Analysis on agarose gels revealed that DNA synthesis in the presence of all four dNTPs took place even if CTP had not been included in the primase reaction and that no DNA synthesis could be detected unless all four dNTPs were present (Supplementary Fig. S6). Thus, for primase displacement by pol III in the G4ori system, a primer elongation by several nucleotides seems to be necessary. However, even when more than 10 nucleotides are added, only one primase molecule per G4ori is displaced. Since binding of primase to G4ori is stabilized by SSB, it is presumably the displacement of SSB by pol III that causes the consequent dissociation of primase.

**Figure 8:**
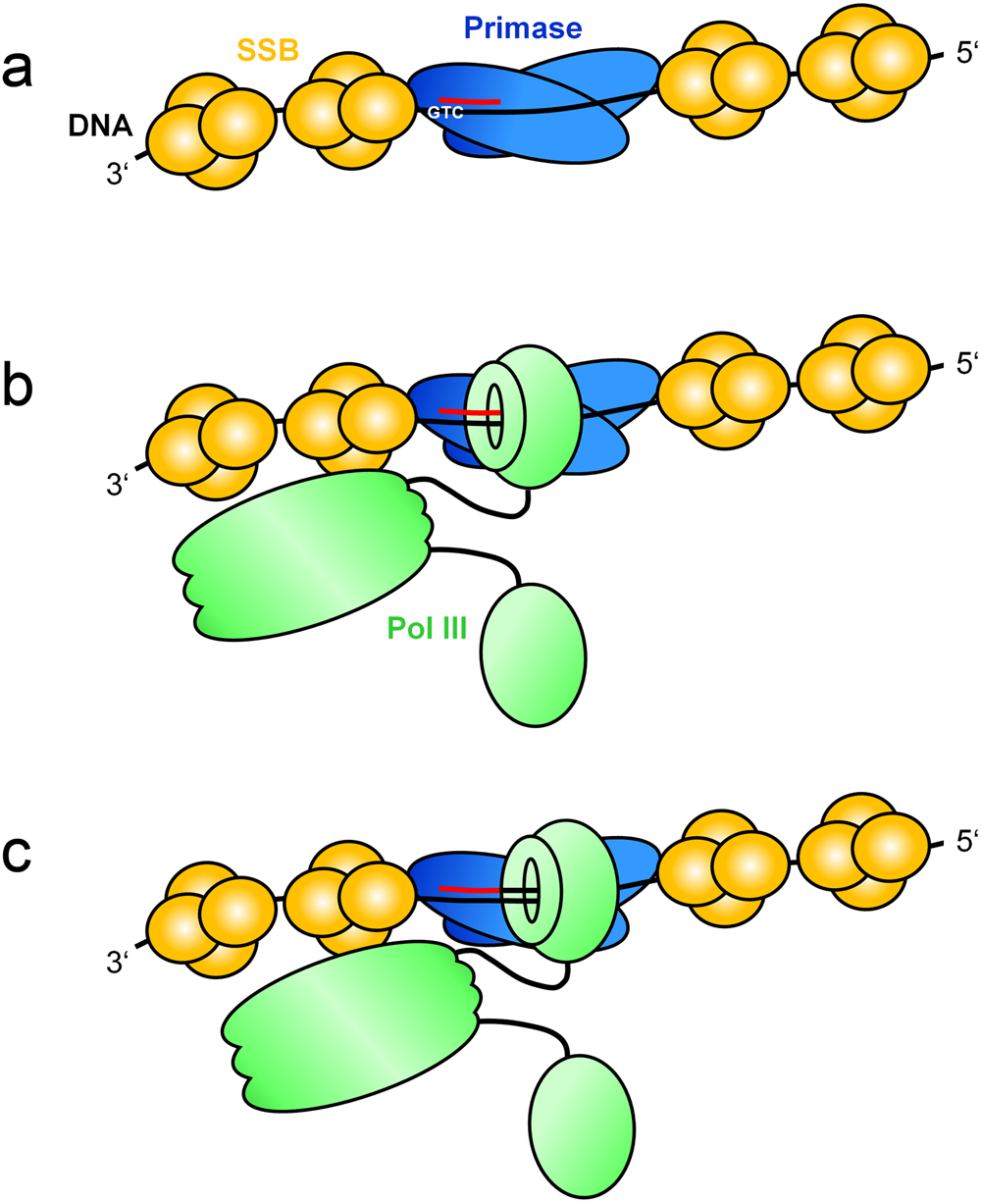
Schematic representation of the primase-to-polymerase switch during DNA replication in *E. coli*. (**a**) Two primase molecules co-operate in the synthesis of the RNA primer (red). (**b**) For elongation of the primer, pol III enters the complex, whereupon primase and pol III are concurrently bound to the primed site, possibly via interactions with the C-termini of an adjacent SSB tetramer. (**c**) During pol III-mediated elongation of the primer by several nucleotides, both enzymes stay bound to the template. Only after the primer has been elongated by more than 10 nucleotides, one of the primases is released in the G4ori system. It is presumably the displacement of SSB by pol III that causes the consequent dissociation of primase. Whereas in the G4ori system the two primases are positioned by hairpin structures that prevent SSB from binding to this part of the origin, at the *E. coli* replication fork primases are brought into contact via their interaction with the replicative helicase DnaB.

The length of the primer synthesized in absence of CTP in our system is comparable to the 10- to 12-nucleotide primer synthesized in chromosomal DNA replication of *E. coli*^25^. Therefore, the observation that a primer elongation of more than 10 nucleotides is necessary in order to displace primase, might also hold true for chromosomal replication (for a schematic representation see Fig. 8). The results obtained by Yuzhakov *et al*.^13^ in the ssM13Gori model system have been generalized to the chromosomal replication fork of *E. coli*, proposing a three-point switch in which primase and the χ subunit of pol III are making mutually exclusive interactions with SSB. Our results, however, show that after primer synthesis, primase and pol III are able to bind concurrently to the primed site, most likely via their interaction with SSB. There is evidence that this might also be the case at the chromosomal replication fork, since the expression of an SSB variant that contains only one C-terminus per tetramer is dominantly lethal *in vivo* and shows defects in coupled leading and lagging strand synthesis *in vitro*^26^. Cells expressing an SSB variant with two C-termini, however, are able to survive and the protein functions equivalently to wild type SSB in coupled leading and lagging strand synthesis^26^. It will be very promising, therefore, to test the impact of these SSB mutants on the primase-to-polymerase switch in our ssM13Gori replication system as a next step.

## Methods

### Determination of DNA and protein concentrations

Protein concentrations were determined using the following extinction coefficients at 280 nm: 113 000 M^−1^cm^−1^ for SSB (tetramer)^27^; 45 840 M^−1^cm^−1^ for DnaG, 125 030 M^−1^cm^−1^ for DNA pol III core, 298 510 M^−1^cm^−1^ for τ clamp loader, and 31 860 M^−1^cm^−1^ for β_2_ clamp as calculated from amino acid composition^28^. Molar concentrations of SSB are given as tetramers. Concentrations of single-stranded M13mp8 and M13Gori DNA (ssM13Gori) were calculated using an extinction coefficient of 8300 M^−1^cm^−1^ per nucleotide, obtained by nuclease digestion of a defined amount of ssM13mp8 followed by determination of the released phosphorus according to Chen *et al*.^29^.

### Preparation of single-stranded M13 DNA

Phage M13mp8^30^ and the respective host *E. coli* JM103 were from Bethesda Research Laboratories (USA). Phage M13Gori^31^ was obtained from ATCC (USA). Single-stranded M13 DNA was isolated from phage particles that were secreted by infected bacterial cells into the surrounding media. ssM13mp8 was purified as described previously^32^.

For the purification of ssM13Gori, an LB culture containing 10 mM MgCl_2_ was inoculated with *E. coli* JM103 cells from an overnight culture. Bacterial cells were grown at 37 °C with constant shaking for 2 h. When OD_600nm_ = 0.1 was reached, the bacterial culture was infected with 0.4 ml sterile filtered M13Gori phages followed by further incubation for 5 h at 37 °C with constant shaking. Bacterial cells were separated from supernatant by centrifugation (5000 × g, 30 min). From M13Gori phage containing supernatant, phage particles were precipitated by addition of 0.6 M NaCl and 6% polyethylene glycol 6000 at 4 °C overnight and subsequent centrifugation (5000 × g, 20 min). Phages were washed by resuspending the pellet in 50 mM Na_2_B_4_O_7_, pH 8.2, precipitated as before, but for 2 h and centrifugation at 6000 × g for 30 min. The phage pellet was stored at −20 °C until further use. To isolate ssM13Gori, the phage pellet was resuspended in a buffer containing 10 mM tris(hydroxymethyl)aminomethane (Tris)/HCl, 3 mM ethylenediaminetetraacetic acid (EDTA), 20 mM NaCl, pH 8.3. In order to lyse the phage particles, a final concentration of 0.5% sodium dodecyl sulfate (SDS) and 0.1 mg/ml proteinase K was added, followed by incubations at 65 °C for 1 h and at 80 °C for 20 min. Afterwards 0.3 M potassium acetate was added to remove viral debris and SDS by centrifugation (6000 × g, 30 min). ssM13Gori containing supernatant was loaded on a Qiagen-Tip 2500 (Qiagen, Germany) and purified according to the QIAGEN Supplementary Protocol “Isolation of single-stranded DNA from M13 phage”. In a last step, ssM13Gori was precipitated using isopropanol, dissolved in water and stored at −20 °C until use.

### Protein purification

Expression and purification of SSB and DnaG were performed as described previously^10,12^. Details regarding the construction of the expression plasmids, the expression in *E. coli* and purification of the subcomplexes of pol III are given in the Supplementary Information. As evident from SDS-PAGE and AUC experiments (Supplementary Fig. S1) the preparations of pol III clamp loader, core and β clamp were of high purity, homogeneous and free of aggregates.

### Fluorescent labelling of DnaG with DyLight488-maleimide

10 mg DnaG were reduced by adding 2 mM tris(2-carboxyethyl)phosphine (TCEP) and incubating at room temperature for 30 min. TCEP was removed by dialysis against 10 mM potassium phosphate, 0.15 M NaCl, pH 7.4. The concentration of DnaG was adjusted to 30 µM and a 3-fold molar excess of DyLight488-maleimide (Thermo Fisher Scientific; 12.5 mM stock in dimethylformamide) was added. The reaction was allowed to proceed for 5-6 h in the dark at 4 °C with slow agitation, followed by an addition of 2 mM dithiothreitol (DTT) to terminate the reaction. Non-reacted dye was partially removed by dialysis against 20 mM Tris/HCl, 0.15 M NaCl, 1 mM TCEP, pH 7.5. Protein solution was concentrated to 500 µl and centrifuged for 20 min at 13 000 × g. To remove residual dye, the protein solution was applied to a Superdex 75 10/300 GL column (GE Healthcare, Germany) and eluted with the same buffer. Fractions containing a high amount of labelled DnaG were pooled, flash-frozen in liquid N_2_ and stored at −80 °C until use. The extinction coefficient of DyLight488 at 493 nm is given as approximately 70 000 M^−1^cm^−1^ (Thermo Fisher Scientific). Concentration of DyLight488-labelled DnaG was determined from absorbance at 280 nm after correction for contributions of the fluorescent dye at this wavelength ${A}_{280nm}^{protein}={A}_{280nm}-0.147\cdot {A}_{493nm}$. UV/Vis spectra of DyLight488-labelled primase were measured in 20 mM Tris/HCl, 150 mM NaCl, 1 mM TCEP at a pH of 7.5 or 9, respectively, and both spectra were virtually identical. The degree of labelling was approximately 1.3 moles of dye per mole protein as calculated from the concentration of dye divided by the concentration of the protein.

To identify the labelling site of the DyLight488 LC-MS analysis using an Orbitrap Fusion Lumos mass spectrometer^33^ coupled to an nanoRSLC system (Thermo Fisher Scientific) was used. Labelled primase was digested with trypsin and analysed by CID and HCD fragmentation techniques. Raw data were searched by Proteome Discoverer (Thermo Fisher Scientific) against an *E. coli* protein data base under standard conditions and a variable DyLight488 modification of cysteine residues inducing a mass shift of 799.74 Da. LC-MS data provided strong evidence that the label is located at the cysteine residue at position 39 (for details see Supplementary Fig. S8). This is in accordance with the previously published observation that primase is site-specifically modified at cysteine 39 when using fluorescein maleimide, which does not lead to any loss in activity, indicating that this residue is not catalytically essential^34^. As our mass spectrometric analysis provided no indication for the modification of a second cysteine, the calculated degree of labelling higher than unity might, at least partially, originate from the fact that the extinction coefficient of the covalent DyLight488-label at 493 nm is not exactly known. However, we cannot rule out that one or several further cysteine residues have been labelled to a minor extend.

We also compared the performance of DyLight488-labelled DnaG and unlabelled DnaG in a DNA replication assay. The assay was performed at the same buffer conditions and using the same protein concentrations as in the AUC experiments (details are given in the Supplementary Information). It can be seen that after approximately 1 min, the major amount of ssM13Gori DNA was converted into the double stranded form, showing that our system is functional with respect to DNA replication (Supplementary Fig. S9).

### Analytical ultracentrifugation (AUC)

Sedimentation velocity experiments were carried out in a ProteomeLab XL-I analytical ultracentrifuge (Beckman Coulter, USA) equipped with a fluorescence detection system (FDS, Aviv Biomedical, USA) using an An-50 Ti rotor. Experiments were performed in AUC sample buffer (20 mM Tris/HCl, 150 mM NaCl, 10 mM MgCl_2_, 1 mM TCEP, pH 7.5) at 20 °C. Analysis of unlabelled samples was performed with the UV/Vis absorption scanning optics at 280 nm using the data acquisition software ProteomeLab XL-I GUI 6.0 (firmware 5.7). Standard 3-mm double-sector centerpieces were filled with 100 µl sample, and were spun at 20 000 rpm. Samples containing DyLight488-labelled DnaG primase were analysed with the FDS using the data acquisition software AOS2. Excitation was performed at 488 nm, emission was detected through a dichroic mirror and a 505–565 nm bandpass filter. Special cell housings (Nanolytics Instruments, Germany) allowed the use of 3-mm standard centerpieces without having to place a spacer between the screw ring and the upper window assembly. Both sectors were filled with 100 µl sample and experiments were performed at 30 000 rpm in the presence of 1 µM bovine serum albumin (BSA) to avoid protein adsorption to surfaces. Data analysis was performed using the program SEDFIT which provides a model for diffusion-deconvoluted differential sedimentation coefficient distributions [c(s) distributions]^35^. All sedimentation coefficients are given as experimental s-values throughout the text. The programme GUSSI was used to prepare the figures representing the AUC data^36^.

To check single-stranded DNA preparations, 20 nM ssM13mp8 or ssM13Gori, respectively, were analysed in AUC experiments using absorbance detection in the absence and presence of 5 µM SSB.

For experiments in the presence of DyLight-labelled primase and pol III, the FDS was used and the AUC samples were composed as follows: 0.8 µM SSB and 5 nM ssM13Gori or 6 nM ssM13mp8 DNA, respectively, were mixed first, followed by the addition of 10 nM DyLight488-DnaG and then 0.1 mM rNTPs. This mixture was incubated at room temperature for 15 min to enable DnaG primase to synthesize the RNA primer. Thereafter, ATP was adjusted to a final concentration of 2 mM and 2.5 to 100 nM pol III (τ_3_δδ‘ψχ: αθε:β_2_=1:3:3) were added. Finally, where indicated, 0.1 mM dNTPs were added. In experiments where ssM13mp8/SSB was used as a control, the concentration of the complex was adjusted to 6 nM to ensure that the same concentration of bound SSB was present as in the case of the somewhat longer ssM13Gori DNA. All experiments were repeated at least 2 to 3 times.

### Fluorescence measurements

Fluorescence measurements were conducted using a Jasco FP-6500 spectrofluorometer. Experiments were performed in AUC sample buffer supplemented with 1 µM BSA at 20 °C. Samples were allowed to thermally equilibrate for 5 min. Excitation and emission bandwidths were set to 3 nm. For the measurement of emission spectra, solutions containing DyLight488-labelled DnaG primase (DL-DnaG) were excited at 488 nm and the emission spectrum was monitored from 488 to 700 nm. Emission spectra of DL-DnaG before and after addition of a 3-fold molar excess of ssM13Gori/SSB or ssM13mp8/SSB, respectively, were recorded in absence and presence of 2 mM ATP and 0.1 mM each of UTP, GTP and CTP. Since the addition of the respective ssDNA/SSB complex resulted in a decrease in the concentration of DL-DnaG from 18 nM to 10 nM, measured fluorescence intensities were corrected for dilution.

For fluorescence titrations, the excitation wavelength was set to 488 nm and the emission intensity was recorded at 520 nm. After each titration step, samples were allowed to equilibrate for 2 min. 10 nM DL-DnaG were titrated with ssM13Gori/SSB up to a concentration of 13 nM (1.6-fold excess of ssM13Gori/SSB over DL-DnaG final). In an inverse titration, 5 nM ssM13Gori/SSB were titrated with DL-DnaG up to a concentration of 13 nM (3-fold excess of DL-DnaG over ssM13Gori/SSB final). Measured fluorescence intensities were corrected for dilution.

### PvuII-cleavage of ssM13Gori

At first, 35 µg ssM13Gori were mixed with a 5-fold molar excess of 5′-GATAAAACAGGTGCCGCTACAGCTGGGGTT-3′ (PvuII site is underlined) in a total volume of 60 µl Buffer G (Thermo Fisher Scientific, USA). Following incubation at 75 °C for 4 min and cooling down to room temperature, 60 units of PvuII were added and the digestion reaction was allowed to proceed for 3.5 hours at 37 °C. Aliquots containing 150 ng of ssM13Gori before and after treatment with PvuII were mixed with the same volume of alkaline sample buffer [30 mM NaOH, 2 mM EDTA, 7% (v/v) Ficoll 400, 0.1% (w/v) SDS, 0.04% (w/v) bromophenol blue]. Electrophoresis was performed on a 1.2%-agarose gel in TBE buffer (0.1 M Tris, 0.1 M boric acid, 2.5 mM EDTA, pH 8.3), the DNA was stained with 1 µg/ml ethidium bromide for 20 min and fluorescence was recorded with an excitation wavelength of 312 nm using a Vilbert Lourmat BIOVISION +  + 1000 fluorescence imaging system.

### Agarose gel electrophoresis of samples after AUC

After AUC experiments, samples were checked for DNA synthesis. To this end, 10 µl sample were treated with 0.5% SDS and 50 µg/ml proteinase K at 56 °C for 30 min, then 0.2 volumes of sample loading buffer [25% (w/v) sucrose, 0.25 M EDTA, 1.2% (w/v) SDS, 0.1% (w/v) bromophenol blue] were added, followed by an incubation at 95 °C for 7 min. Electrophoresis was performed on a 1%-agarose gel in TPE buffer (89 mM Tris/H_3_PO_4_, 2 mM EDTA, pH 8.0). Afterwards, the DNA was stained for 40 min with SYBR Gold (Invitrogen, USA) diluted 1:10 000 in TE buffer. Fluorescence was detected as described above.

## Supplementary information

Supplementary Information
