# Supplementary material for: E. coli primase and DNA polymerase III holoenzyme are able to bind concurrently to a primed template during DNA replication
Source: Sci Rep. 2019 Oct 8;9:14460. doi: 10.1038/s41598-019-51031-0 (PMC6783573; doi:10.1038/s41598-019-51031-0)
Supplement: Supplementary file 1 — Supplementary Information [file 41598_2019_51031_MOESM1_ESM.pdf]

SUPPLEMENTARY INFORMATION

E. coli primase and DNA polymerase III holoenzyme are able to bind concurrently to a primed template during DNA replication

Andrea Bogutzki[1], Natalie Naue[1,3], Lidia Litz[1], Andreas Pich[2] and Ute Curth[1*]

[1]Institute for Biophysical Chemistry, Hannover Medical School, Hannover, 30625, Carl-Neuberg-Str. 1, Germany

[2]Institute for Toxicology, Hannover Medical School, Hannover, 30625, Carl-Neuberg-Str. 1, Germany

[*]Correspondence and requests for materials should be addressed to U.C. (email: curth.ute@mh-hannover.de)

[3]Present Address: Inamed GmbH, Gauting, 82131, Germany

Generation of expression plasmids encoding for DNA polymerase III holoenzyme

Restriction endonucleases, Phusion High-Fidelity DNA Polymerase, Pfu DNA Polymerase and T4 DNA Ligase were obtained from NEB (Germany), Fermentas (Germany) or Thermo Scientific (Germany) and were used according to the manufacturer's protocol. Oligonucleotides (MWG Biotechnologies, Germany) used to clone the constructs in this study are given below. Expression vector pETM11-SUMO3GFP and the plasmid pETM11-SenP2, carrying the gene for the SUMO-specific protease, were obtained from EMBL Protein Expression and Purification Core Facility (Germany). pETDuet-1, pRSFDuet-1, pACYCDuet-1 and pCDFDuet-1 were purchased from Novagen (Germany). Genes were amplified by PCR from genomic *E. coli* LK111(λ) DNA[1] using a proof-reading polymerase and cloned into the respective expression plasmids using endonuclease restriction followed by ligation using T4 DNA ligase. Plasmid DNA was amplified and isolated using standard protocols. Recombinant DNA was transformed into chemically competent *E. coli* cells according to the TSS-method[2]. All recombinant plasmids were sequenced to verify the sequence of the inserted genes (GATC, Germany). *E. coli* DH5α and LK111(λ) were used for cloning and amplification of plasmid DNA.

Relevant information about primers used for cloning and the generated plasmids are given below. Start and stop codons are marked in bold, restriction enzymes used for cloning are given and their restriction sites are underlined.

Expression plasmid	Primer	Use	Primer sequence (5′ to 3′)
pETM11-SUMO3-α	alpha_fwd	Amplification of *dnaE gene*	AGGAGGTCTCACCGGTGGA**ATG**TCTGAACCACGTTTCGT (BsaI)
	alpha_rev		AGGAGAGCTC**TTA**GTCAAACTCCAGTTCCAC (SacI)
pETM11-SUMO3-ε	epsilon_fwd	Amplificatin of *dnaQ* gene	AGGACCCGGGGGA**ATG**AGCACTGCAATTACAC (XmaI)
	epsilon_rev		AGGACTCGAG**TTA**TGCTCGCCAGAGGCAACT (XhoI)
pRSFDuet-θ	theta_fwd	Amplification of *holE* gene	AGGAGGTCTCGC**ATG**CTGAAGAATCTGGCTAAA (BsaI)
	theta_rev		AGGAGGATCC**TTA**TTTAAGTTTGGGCTCGTAAGG (BamHI)
pRSFDuet-β	beta_fwd	Amplification of *dnaN* gene	CTAGGACAT**ATG**AAATTTACCGTAGAACGT (NdeI)
	beta_rev		AGGAGGTACC**TCA**CAGTCTCATTGGCATGACAAC (KpnI)
pRSFDuet-τ	tau_fwd	Amplification of *dnaX* gene	AGGAGGTCTCGC**ATG**AGTTATCAGGTCTTAGCC (BsaI)
	tau_rev		AGGAGCGGCCGC**TCA**AATGGGGCGGATACTTTCTTC (NotI)
	tau_mut_fwd	Removal of frame shift (mutagenesis)	GGAGCAACCAAAGCAAAGAAGAGCGAACCGGCAGCCGCT (SapI)
	tau_mut_rev		AGCGGCTGCCGGTTCGCTCTTCTTTGCTTTGGTTGCTCC (SapI)
pETDuet-$\chi\psi$	psi_fwd	Amplification of *holD* gene	AGGAGGTCTCGC**ATG**ACATCCCGACGAGACTGG (BsaI)
	psi_rev		AGGAGCGGCCGC**TCA**GTCGTTTCGAGGGAAGAAATC (NotI)
	chi_fwd	Amplification of *holC* gene	AGGACAT**ATG**AAAAACGCGACGTTCTACCTTCTG (NdeI)
	chi_rev		AGGACTCGAG**TCA**TTTCCAGGTTGCCGT (XhoI)
pCDFDuet-CmR-$\delta'\delta$	delta_fwd	Amplification of *holA* gene	AGGACAT**ATG**ATTCGGTTGTACCCGGAA (NdeI)
	delta_rev		AGGACTCGAGTCAACCGTCGATAAATACGTC (XhoI)
	delta_prime_fwd	Amplification of *holB* gene	AGGAGGTCTCGC**ATG**AGATGGTATCCATGGTTA (BsaI)
	delta_prime_rev		AGGAAAGCT**TCA**AAGATGAGGAACCGGTAGCAC (HindIII)

Cloning of the genes encoding DNA polymerase III core (subunit composition αεθ). The amplified gene encoding α (*dnaE*) was digested using BsaI and SacI and inserted into AgeI/SacI-treated pETM11-SUMO3GFP, yielding pETM11-SUMO3-α. To generate pETM11-SUMO3-ε, the amplified gene encoding ε (*dnaQ*) was digested with XmaI and XhoI and inserted into AgeI/XhoI-treated pETM11-SUMO3GFP. The gene encoding θ (*holE*) was digested with BsaI and BamHI and cloned into NcoI/BamHI treated vector pRSFDuet-1, yielding pRSFDuet-θ.

Cloning of the gene encoding the β_2 clamp. The gene encoding the β_2 clamp (*dnaN*) was cloned into pRSF-Duet-1 using the restriction endonucleases NdeI and KpnI, yielding pRSFDuet-β.

Cloning of the genes encoding the τ clamp loader (subunit composition $\tau_3\delta\delta'\chi\psi$). The amplified gene encoding τ (*dnaX*) was digested using BsaI and NotI and inserted into NcoI/NotI-treated pRSFDuet-1, yielding pRSFDuet-τ-FS. The gene encoding τ contains a frameshift site that frequently leads to a premature stop of gene expression by a -1 ribosomal frameshift resulting in the expression of the γ isoform[3,4]. To circumvent this translational frameshift, two point mutations that do not change the amino acid sequence of the translated gene product and additionally create a SapI restriction site were introduced by site-directed mutagenesis. The final plasmid pRSFDuet-τ contains the τ gene without the translational frameshift site.

The genes encoding the χ (*holC*) and ψ (*holD*) subunits were cloned into pETDuet-1 in two steps. The amplified gene encoding χ was digested using BsaI and NotI and inserted into NcoI/NotI-treated pETDuet-1. Then, the gene encoding ψ was inserted into this vector by using the restriction endonucleases NdeI and XhoI. The final construct pETDuet-χψ contains the genes for χ and ψ.

The genes encoding the δ (*holA*) and δ′ (*holB*) subunits were cloned into pCDFDuet-1 in two steps. The gene encoding δ was inserted using the restriction endonucleases NdeI and XhoI. After digestion with BsaI and HindIII, the gene encoding δ′ was cloned into this vector treated with NcoI/HindIII, yielding pCDFDuet-δ′δ. To generate pCDFDuet-CmR-δ′δ, the δ′δ encoding fragment of pCDFDuet-δ′δ obtained by EcoNI and Eco0109I digestion was inserted into pCDFDuet-CmR digested with the same restriction enzymes. pCDFDuet-CmR was generated by replacing the gene conferring streptomycin resistance of pCDF-Duet-1 by the gene conferring chloramphenicol resistance of pACYCDuet-1 using DrdI and NheI digestion. The final plasmid pCDFDuet-CmR-δ′δ contains the genes encoding δ and δ′ and the gene conferring chloramphenicol resistance.

Expression of recombinant genes

Expression strains were generated by transformation of the respective recombinant plasmid(s) into *E. coli* DE3 strains as given below. Arctic Express DE3 (Novagen, Germany), Rosetta DE3 pLysS (Novagen, Germany) and BT282 (AB1157 *xonA2 endA::Tet*) DE3 pLysS were used as expression strains.

Relevant information and antibiotic resistance (Tet = tetracycline, Kan = kanamycin, Amp = ampicillin, Cm = chloramphenicol) of the expression strains used in this study are given below.

***E. coli* strain**	**Plasmids**	**Product**	**Antibiotics**
Arctic Express DE3	pETM11-SUMO3-α	α subunit	30 µg/ml Cm, 15 µg/ml Kan
BT282 DE3 pLysS	pETM11-SUMO3-ε	ε subunit	15 µg/ml Tet, 30 µg/ml Cm, 15 µg/ml Kan
BT282 DE3 pLysS	pRSFDuet-θ	θ subunit	15 µg/ml Tet, 30 µg/ml Cm, 15 µg/ml Kan
Rosetta DE3 pLysS	pRSFDuet-β	β_2 clamp	30 µg/ml Cm, 15 µg/ml Kan
BT282 DE3	pRSFDuet-τ, pETDuet-χψ, pCDFDuet-CmR-δ′δ	τ clamp loader	100 µg/ml Amp, 30 µg/ml Cm, 15 µg/ml Kan

Overnight cultures of bacterial strains were grown in Luria Bertani (LB) medium (10 g/l casein, 5 g/l yeast extract, 10 g/l NaCl) supplemented with the respective antibiotics and 1% (w/v) glucose. If not stated otherwise, gene expression was performed in the absence of antibiotics and glucose.

Purification of the subcomplexes of DNA polymerase III

All purifications steps were carried out at 4°C, protein solutions were cleared by centrifugation (100 000 x g, 45 min) before applying them to chromatography columns. For size exclusion chromatography (SEC), protein solutions were sterile-filtered before loading. Purified proteins were flash-frozen in liquid nitrogen in aliquots of up to 0.1 ml and stored at -80°C until use.

The following buffers were used for the purification of DNA Pol III core:
Buffer A: 20 mM potassium phosphate, 10% (v/v) glycerol, 0.3 M NaCl, 5 mM 2-mercaptoethanol (2-ME), pH 7.4
Buffer B: Buffer A containing 0.05 M NaCl
Buffer C: Buffer A containing 0.5 M NaCl

The following buffers were used for the purification of τ clamp loader and β_2 clamp:
Buffer D: 50 mM tris(hydroxymethyl)aminomethane (Tris)/HCl, 10% (v/v) glycerol,
1 mM ethylenediaminetetraacetic acid (EDTA), 1 or 5 mM dithiothreitol (DTT), pH 7.5
Buffer E: 20 mM potassium phosphate, 10% (v/v) glycerol, 1 mM DTT, pH 7.2
Buffer F: 20 mM Tris/HCl, 10% (v/v) glycerol, 50 mM NaCl, 5 mM DTT, pH 7.5
Buffer G: 20 mM potassium phosphate, 10% (v/v) glycerol, 0.3 M NaCl, 1 or 5 mM DTT, pH 7.5
For purification of β_2 clamp or τ clamp loader, buffers were supplemented with 1 mM or 5 mM DTT respectively.

Gene expression and cell disruption. *E. coli* cultures were grown in LB medium and gene expression was induced by addition of 1 mM IPTG. Rosetta DE3 pLysS/pRSFDuet-β was grown at 37°C and gene expression was induced at OD_{600nm}=1.5 for 3 h. Arctic Express DE3/pETM11-SUMO3-α was grown at 30°C until OD_{600nm}=1. After cooling to 13°C, gene expression was induced for 24 h at 13°C. *E. coli* BT282 DE3 pLysS/pETM11-SUMO3-ε and *E. coli* BT282 DE3 pLysS/pRSFDuet-θ were grown at 37°C and gene expression was induced for 5 h. BT282 DE3/pRSFDuet-τ, pCDFDuet-CmR-δ′δ, pETDuet-χψ was grown in presence of 0.2 g/l kanamycin and 2 g/l glucose at 37°C and gene expression was induced at OD_{600nm}=1.0 for 5 h.

Following gene expression, bacterial cells were harvested by centrifugation (15 min, 5000 x g) and resuspended in 1 ml buffer per 1 g of bacterial cells. For Rosetta DE3 pLysS/pRSFDuet-β and BT282 DE3/pRSFDuet-τ, pCDFDuet-CmR-δ′δ, pETDuet-χψ, 50 mM Tris/HCl, 10% sucrose, pH 7.5 was used as resuspension buffer. Arctic Express DE3/pETM11-SUMO3-α, BT282 DE3 pLysS/pETM11-SUMO3-ε and BT282 DE3 pLysS/pRSFDuet-θ were resuspended in 0.14 M NaCl, 2.7 mM KCl, 10 mM Na_2HPO_4, 1.8 mM KH_2PO_4, pH 7.3. Resuspended bacterial cells were flash-frozen in liquid N_2 and stored at -80°C until use.

For protein purification, the cell suspension was thawed on ice in the presence of 0.1 mM phenylmethylsulfonyl fluoride (PMSF), 5 mM benzamidine, 0.5 mM DTT and 1 tablet of "Complete EDTA free Protease Inhibitor Cocktail Tablets" (Roche Diagnostics, Germany) per 50 ml suspension. Bacterial cells were lysed by sonication, followed by centrifugation (100 000 x g for 45 min) to remove cell debris. Unless otherwise specified, chromatography columns were equilibrated with the buffer in which the sample was applied to the respective column. For affinity purification of His-tagged proteins, Protino Ni-NTA Agarose (Machery-Nagel, Germany) was used as column material. HiLoad 26/60 Superdex 200 PG columns (GE Healthcare, Germany) were used for size exclusion chromatography (SEC), unless stated otherwise.

Purification of DNA polymerase III core. To purify the α subunit of DNA Pol III, the frozen cell suspension was diluted with one volume of buffer A + 20 mM imidazole supplemented with the components described above. Following cell disruption, His_6-SUMO3-α containing supernatant was loaded on Ni-NTA agarose equilibrated with buffer A + 20 mM imidazole. The column was washed using 2 column volumes (CV) of buffer A + 40 mM imidazole followed by elution with buffer A + 0.3 M imidazole. Fractions containing a high amount of His_6-SUMO3-α were pooled. During dialysis against buffer B, 0.5 µg SenP2 (SUMO-specific protease) per mg protein were added for at least 8 h to remove the His_6-SUMO3-tag and the sample was loaded on a Q Sepharose Fast Flow column (Amersham Biosciences, United Kingdom). The column was washed using 2 CV of buffer B and a linear gradient from 0.050 to 0.5 M NaCl in buffer B was used for elution. Fractions containing a high amount of α were pooled, concentrated to 10 ml and applied to SEC in buffer A. Proteins were eluted using the same buffer and fractions containing a high amount of α were pooled.

His_6-SUMO3-ε expressed on its own is insoluble. Therefore, this protein was purified using a protein denaturation and refolding protocol based on[5]. The frozen cell suspension was diluted with two volumes of buffer C supplemented with the components described above. Following cell disruption, His_6-SUMO3-ε remained associated with the cell debris pellet after centrifugation. The pellet was resuspended using buffer C and homogenised using sonication. After centrifugation (100 000 x g, 45 min), the pellet was resuspended in buffer C + 3 M guanidinium hydrochloride (GuaHCl), centrifuged as before and the His_6-SUMO3-ε containing supernatant was dialysed against the 10-fold volume of buffer C. The buffer was changed 3 times and for the first dialysis step the stirrer was turned off for the first 2–3 h to ensure a slow decrease of GuaHCl concentration. Afterwards the protein solution was cleared using centrifugation (100 000 x g, 45 min) and His_6-SUMO3-ε was precipitated using 0.35 g/ml $(NH_4)_2SO_4$. The pellet was resuspended in buffer A + 20 mM imidazole and dialysed against the same buffer.

To reconstitute the εθ subcomplex, refolded His_6-SUMO3-ε was mixed with a θ containing cell lysate. For this, the frozen cell suspension was diluted with two volumes of buffer A + 20 mM imidazole supplemented with the components described above. Following cell disruption, the θ containing supernatant was mixed with refolded His_6-SUMO3-ε and incubated for 10 min. The protein solution was applied to a Ni-NTA agarose column equilibrated with buffer A + 20 mM imidazole. The column was washed using 2 CV of buffer A + 40 mM imidazole followed by elution with buffer A + 0.3 M imidazole. Fractions containing a high amount of His_6-SUMO3-εθ subcomplex were pooled and dialysed against buffer A + 20 mM imidazole.

To reconstitute DNA Pol III core complex (subunit composition αεθ), purified α subunit was mixed with His_6-SUMO3-εθ subcomplex. The protein solution was incubated for 10 min and applied to Ni-NTA agarose equilibrated with buffer A + 20 mM imidazole. The column was washed using 2 CV of buffer A + 40 mM imidazole followed by elution with buffer A + 0.3 M imidazole. Fractions containing a high amount of His_6-SUMO3-αεθ complex were pooled and 80 µg SenP2 per mg protein were added and incubated for 1 h to remove the His_6-SUMO3 tag. Then, the protein solution was applied to SEC in buffer A. Proteins were eluted using the same buffer and fractions containing a high amount of αεθ were pooled and stored as described above.

Purification of the β_2 clamp. Purification of the β_2 clamp was performed as described in Johanson et al.[6] with some exceptions. Following cell disruption, the β_2 clamp was further purified by fractionated $(NH_4)_2SO_4$ precipitation (0.226 g/ml and 0.413 g/ml). The pellet of the second $(NH_4)_2SO_4$ precipitation was resuspended in buffer D and dialysed against the same buffer. The protein solution was applied to a Q Sepharose Fast Flow column (Amersham Biosciences, UK) and proteins were eluted using a linear gradient (15 CV) from 0 to 0.3 M NaCl in buffer D. Fractions containing a high amount of β_2 clamp were pooled and precipitated using 0.413 g/ml $(NH_4)_2SO_4$. The pellet was resuspended in buffer E and dialysed against the same buffer. The protein solution was applied to a Macro-Prep Ceramic Hydroxyapatite Type I 20 µm (Bio-Rad, USA) column and the β_2 clamp containing flow through was collected, concentrated to

10 ml and applied to a HiLoad 26/60 Superdex 75 PG column (GE Healthcare, Germany) equilibrated with buffer G. Proteins were eluted using the same buffer and fractions containing a high amount of β_2 clamp were pooled and stored as described above.

Purification of the τ clamp loader. The frozen cell suspension was diluted with one volume of 50 mM Tris/HCl, 10% (w/v) sucrose, 0.1 M NaCl, 15 mM spermidine, 1 mM EDTA, pH 7.5 supplemented with the components described above. Centrifugation steps were performed for 25 min at 50 000 x g. Following cell lysis, τ clamp loader containing supernatant was precipitated using 0.2 g/ml $(NH_4)_2SO_4$ and the pellet was washed with 50 mM Tris, 20% (v/v) glycerol, 100 mM NaCl, 0.15 g/ml $(NH_4)_2SO_4$, 1 mM EDTA and 5 mM DTT. Following centrifugation, the pellet was resuspended in buffer F and precipitated as before. After centrifugation the pellet was resuspended in buffer F and conductivity was adjusted to 5.82 mS/cm by addition of the same buffer but omitting NaCl. Then, the protein solution was loaded on a Heparin Sepharose 6 Fast Flow column (GE Healthcare, UK) equilibrated with buffer F. The column was washed using the same buffer (15 CV) and proteins were eluted by a linear gradient (10 CV) from 0.050 to 0.5 M NaCl in buffer F. Fractions containing a high amount of τ clamp loader were pooled and precipitated using 0.25 mg/ml $(NH_4)_2SO_4$. Following centrifugation, the pellet was resuspended in buffer D + 0.1 M NaCl and conductivity was adjusted to 5.82 mS/cm. The protein solution was applied to a SP Sepharose Fast Flow (GE Healthcare, UK) column equilibrated with buffer D + 0.1 M NaCl. The column was washed using the same buffer (3 CV) and proteins were eluted by a linear gradient (8 CV) from 0.1 to 0.3 M NaCl in buffer D. Fractions containing a high amount of τ clamp loader were pooled and precipitated using 0.25 mg/ml $(NH_4)_2SO_4$. Following centrifugation, the pellet was resuspended in buffer G and the protein solution was applied to SEC. Proteins were eluted using the same buffer and fractions containing a high amount of τ clamp loader were pooled and stored as described above.

Preparation of DNA polymerase III holoenzyme. Pol III was prepared by mixing of τ clamp loader with a 3-fold molar excess of core and β_2 clamp in AUC sample buffer. The concentrations of pol III given throughout the text correspond to pol III holoenzyme with a subunit composition of $(\tau_3\delta\delta'\chi\psi)$ $(\alpha\epsilon\theta)_3$ $(\beta_2)_3$.

Purified sub-complexes of pol III were analysed using SDS-PAGE and AUC (Supplementary Fig. S1).

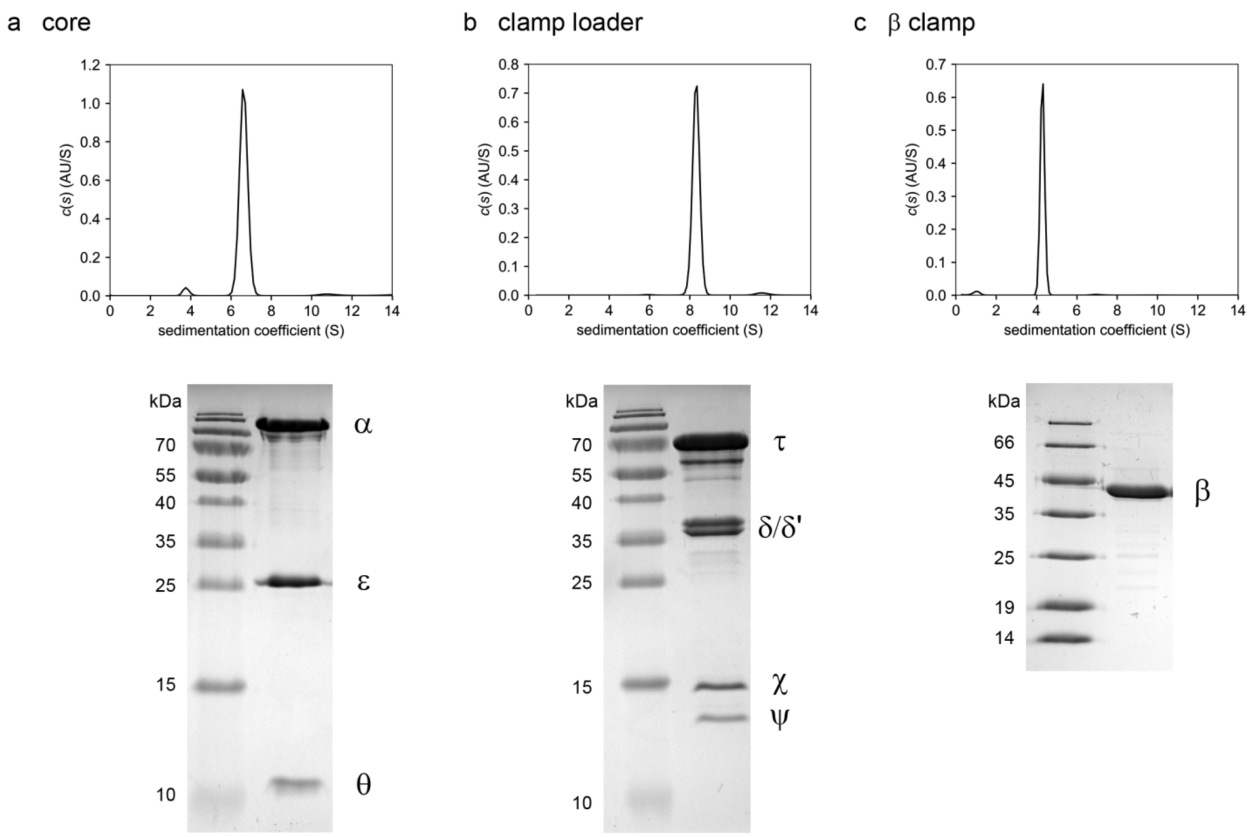

Supplementary Figure S1: Analysis of the purified sub-complexes of pol III holoenzyme by SDS-PAGE and AUC. (**a**) core, (**b**) clamp loader, (**c**) β_2 clamp. 10 µg of core (subunit composition $\alpha\varepsilon\theta$), 10 µg of clamp loader (subunit composition $\tau_3\delta\delta'\chi\psi$) and 2 µg β_2 clamp were analysed on a 12% SDS-PAGE followed by Coomassie staining. The individual subunits of pol III and the size of the bands of the protein molecular weight marker are indicated. Sedimentation velocity experiments were performed at 20°C in 20 mM potassium phosphate, 300 mM NaCl, 0.5 mM DTT, pH 7.4 for β_2 clamp and 20 mM Tris/HCl, 150 mM NaCl, 10 mM $MgCl_2$, 1 mM TCEP, pH 7.5 for core and clamp loader. The sedimentation of 2 µM core at 30 000 rpm was detected at 230 nm and the sedimentation of 1 µM clamp loader or 5 µM β_2 clamp at 50 000 rpm was detected at 280 nm. *c*(*s*) distributions were obtained using the programme package SEDFIT and experimental s-values are given.

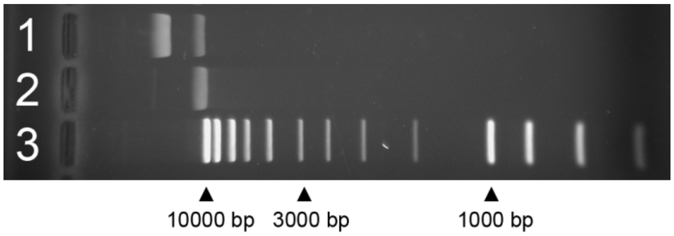

Supplementary Figure S2: Analysis of ssM13Gori in alkaline sample buffer on a 1.2%-agarose gel in TBE before and after annealing of a PvuII-site containing oligonucleotide and PvuII cleavage.

Lane 1: ssM13Gori; lane 2: ssM13Gori after PvuII restriction; lane 3: Molecular weight marker (Hyperladder I, Bioline, UK) providing double-stranded DNA fragments of 200, 400, 600, 800, 1000, 1500, 2000, 2500, 3000, 4000, 5000, 6000, 8000 and 10000 bp.

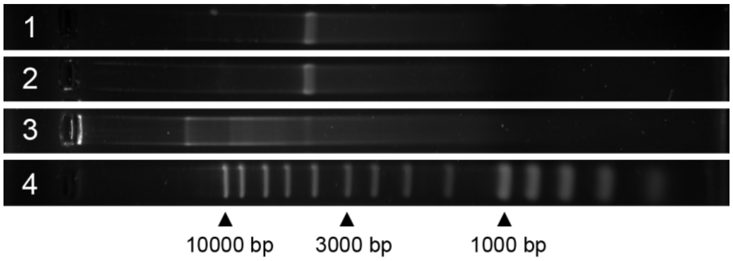

Supplementary Figure S3: Analysis of samples after AUC, similar to those shown in Fig. 3c, on a 1%-agarose gel run in TPE. All four rNTPs were used during primer synthesis. DNA synthesis was performed in the presence of 0.1 mM of all four dNTPs. In the presence of dNTPs, DNA synthesis was observed.

Lane 1: 5 nM ssM13Gori/SSB/primase complex in the presence of 2 mM ATP and 0.1 mM each of UTP, GTP and CTP; lane 2: as in lane 1 with 15 nM pol III added; lane 3: as in lane 1 with 15 nM pol III and all four dNTPs added; lane 4: Molecular weight marker (Hyperladder I, Bioline, UK) providing double-stranded DNA fragments of 200, 400, 600, 800, 1000, 1500, 2000, 2500, 3000, 4000, 5000, 6000, 8000 and 10000 bp. All lanes were taken from a single gel picture. For reasons of clarity interjacent lanes were removed.

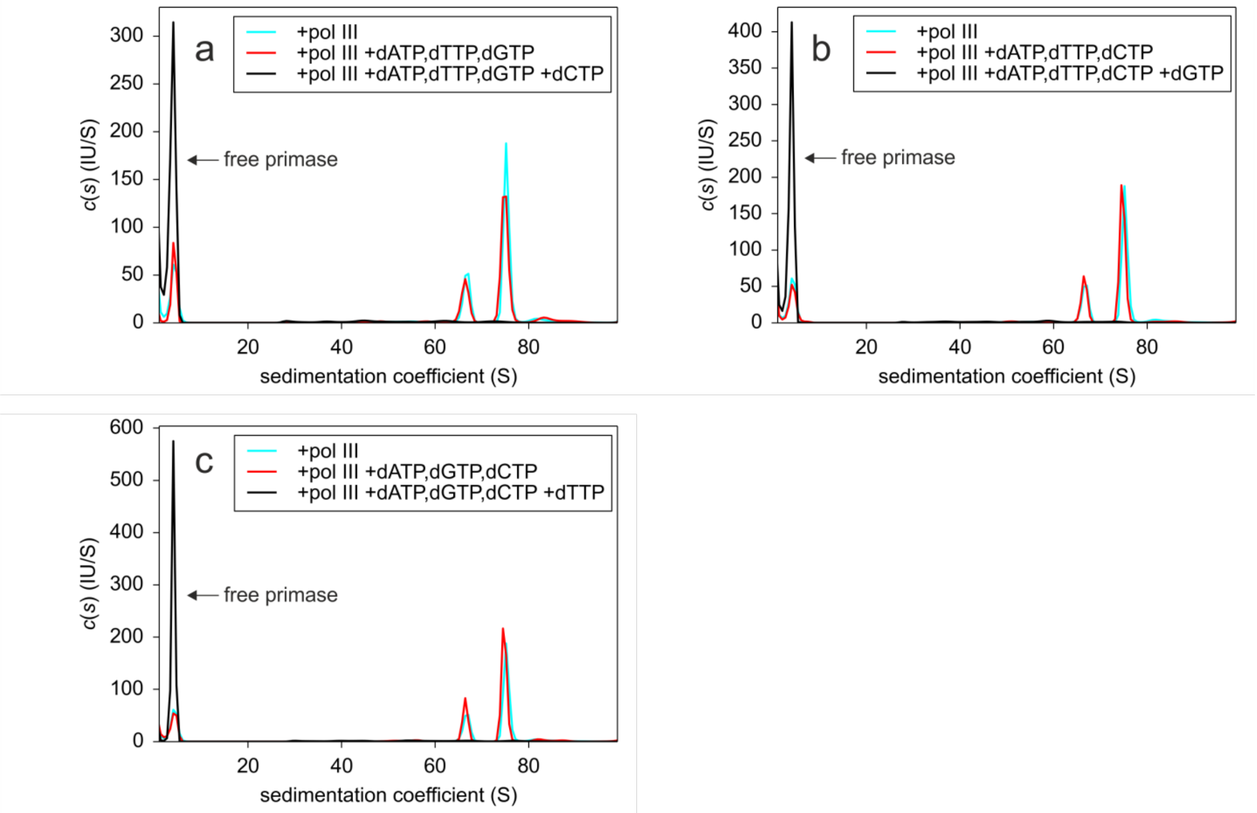

Supplementary Figure S4: Effect of the addition of different mixtures of three dNTPs on the displacement of primase from ssM13Gori/SSB by pol III after primer synthesis in the absence of CTP. A complex of 5 nM ssM13Gori/SSB/DL-DnaG in the presence of 2 mM ATP and 0.1 mM each of UTP and GTP was incubated with 15 nM pol III in the absence (cyan) and presence (red) of 0.1 mM each of a mixture of (**a**) dATP, dTTP, dGTP (**b**) dATP, dTTP, dCTP or (**c**) dATP, dGTP, dCTP. As a control, 0.1 mM of the respective missing fourth dNTP was added to the mixtures (black). Whereas the addition of the different mixtures of three dNTPs in the presence of pol III did not result in a significant release of primase from the ssDNA/SSB complex, further addition of the respective missing fourth dNTP resulted in a complete displacement of primase in all cases, showing that the system was fully functional.

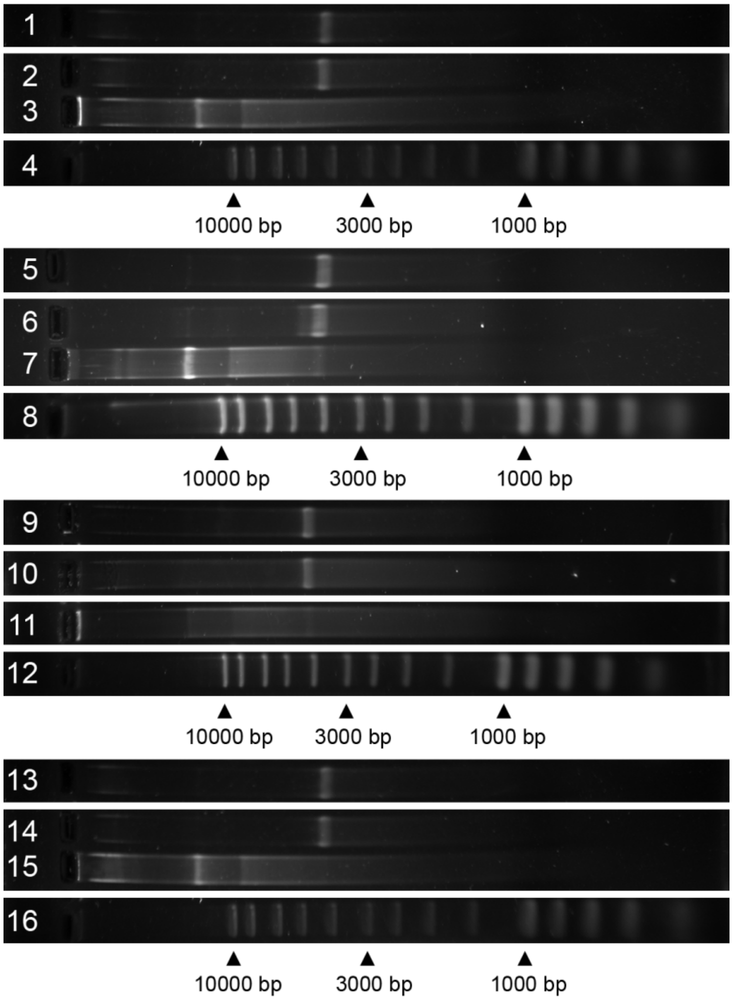

Supplementary Figure S5: Analysis of the samples shown in Fig. 6a-d after AUC on a 1%-agarose gel run in TPE. All four rNTPs were used during primer synthesis. DNA synthesis was performed in the presence of 0.1 mM of the indicated combinations of three dNTPs. To check whether all components were present to allow for DNA synthesis, 0.1 mM of the missing fourth dNTP was added as a control in a second sample. Detectable DNA synthesis was only observed in the presence of all four dNTPs.

Lanes 1, 5, 9, 13: 5 nM ssM13Gori/SSB/primase complex in the presence of 2 mM ATP and 0.1 mM each of UTP, GTP and CTP as well as 15 nM pol III; lane 2: as in lane 1 with dATP, dTTP and dGTP added; lane 3: as in lane 2 with additional dCTP; lane 6: as in lane 5 with dATP, dTTP and dCTP added; lane 7: as in lane 6 with additional dGTP; lane 10: as in lane 9 with dATP, dGTP and dCTP added; lane 11: as in lane 10 with additional dTTP; lane 14: as in lane 13 with dTTP, dGTP and dCTP added; lane 15: as in lane 14 with additional dATP; lanes 4, 8, 12 and 16: Molecular weight marker (Hyperladder I, Bioline, UK) providing double-stranded DNA fragments of 200, 400, 600, 800, 1000, 1500, 2000, 2500, 3000, 4000, 5000, 6000, 8000 and 10000 bp. Lanes 1-4 and 13-16 were taken from a single picture of one gel, lanes 5-8 from a picture of a second gel and lanes 9-12 from a third one. For reasons of clarity interjacent lanes were removed.

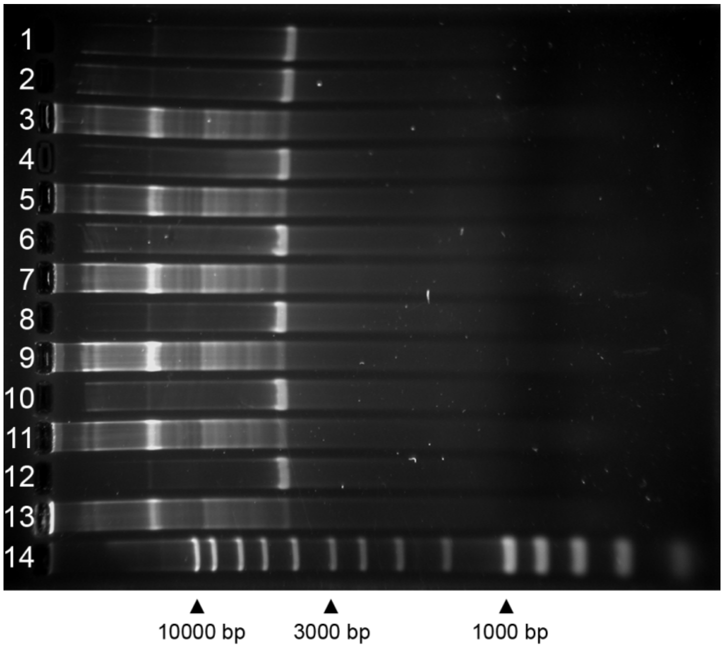

Supplementary Figure S6: Analysis of the samples shown in Fig. 7a and b and Supplementary Fig. S4a-c after AUC on a 1%-agarose gel run in TPE. CTP was omitted during primer synthesis. DNA synthesis was performed in the presence of 0.1 mM of the indicated combinations of dNTPs. To check whether all components were present to allow for DNA synthesis, 0.1 mM of the missing dNTPs were added as a control in a second sample. Detectable DNA synthesis was only observed in the presence of all four dNTPs.

Lane 1: 5 nM ssM13Gori/SSB/primase complex in the presence of 2 mM ATP and 0.1 mM each of UTP and GTP; lane 2: as in lane 1 with 15 nM pol III added; lane 3: as in lane 2 with addition of all four dNTPs; lane 4: as in lane 2 with dTTP, dGTP and dCTP added; lane 5: as in lane 4 with additional dATP; lane 6: as in lane 2 with dATP, dTTP and dCTP added; lane 7: as in lane 6 with additional dGTP; lane 8: as in lane 2 with dATP, dGTP and dCTP added; lane 9: as in lane 8 with additional dTTP; lane 10: as in lane 2 with dATP, dTTP and dGTP added; lane 11: as in lane 10 with additional dCTP; lane 12: as in lane 2 with dGTP and dCTP added; lane 13: as in lane 12 with additional dATP and dTTP; lane 14: Molecular weight marker (Hyperladder I, Bioline) providing double-stranded DNA fragments of 200, 400, 600, 800, 1000, 1500, 2000, 2500, 3000, 4000, 5000, 6000, 8000 and 10000 bp.

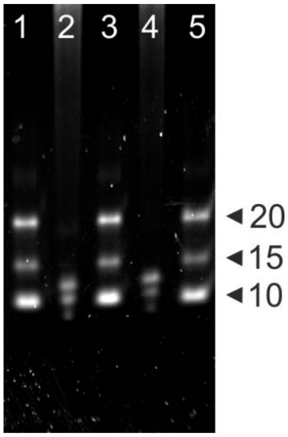

Supplementary Figure S7: Length of the primers synthesized by DnaG or DL-DnaG in the absence of CTP. Lane 2: primer synthesis by DnaG as described below, lane 4: primer synthesis by DL-DnaG as described below; lane 1, 3, 5: DNA oligonucleotides of the indicated lengths were used as a reference: 20 nucleotides (5 ng), 15 nucleotides (5 ng), 10 nucleotides (15 ng).

Experiments were performed in AUC sample buffer using the same concentrations and reaction conditions as in the AUC experiments: 0.8 µM SSB and 5 nM ssM13Gori were mixed first, followed by the addition of 10 nM DnaG or DL-DnaG. Primer synthesis was performed in presence of 0.1 mM each of ATP, UTP and GTP for 15 min at room temperature. Afterwards 0.9 ml of each sample were treated with 55 µg/ml proteinase K and 0.55% SDS for 1 h at 65°C. To facilitate precipitation with 73% ethanol, 50 µg glycogen and sodium acetate to a final concentration of 0.3 M were added. After centrifugation the pellet was vacuum-dried, washed with 70% ethanol, again vacuum-dried and dissolved in 10 µl 95% formamide, 10 mM EDTA, 0.1 mg/ml bromophenol blue. After incubation for 5 min at 65°C, samples were run on a 17% PAGE, 7 M urea in TBE buffer and the nucleic acids were stained for 20 min with SYBR Gold (Invitrogen, USA) diluted 1:10 000 in TE buffer. Fluorescence was detected as described in the Methods section.

a

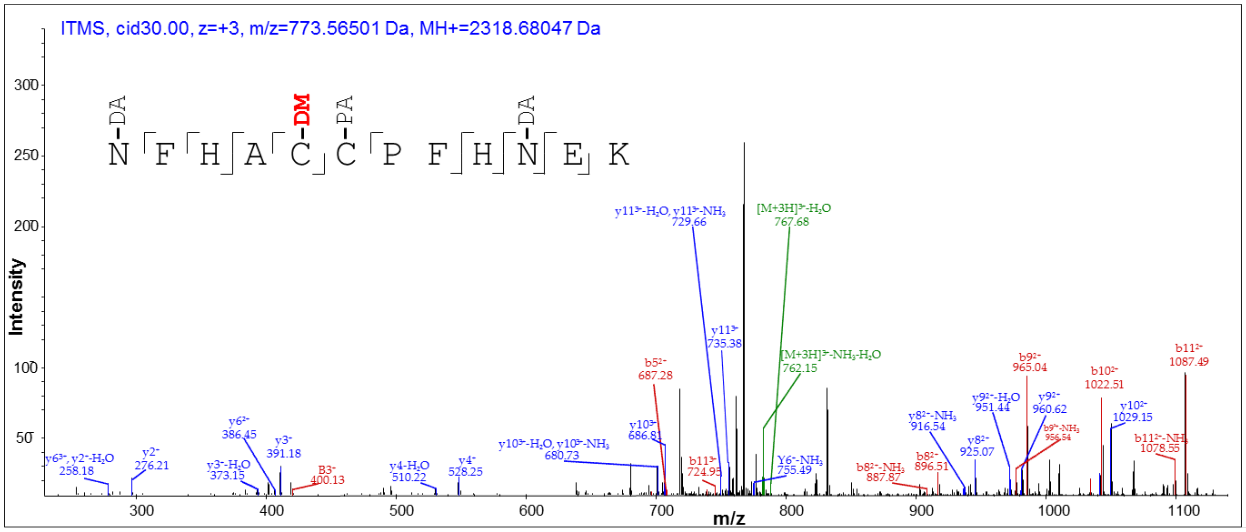

b

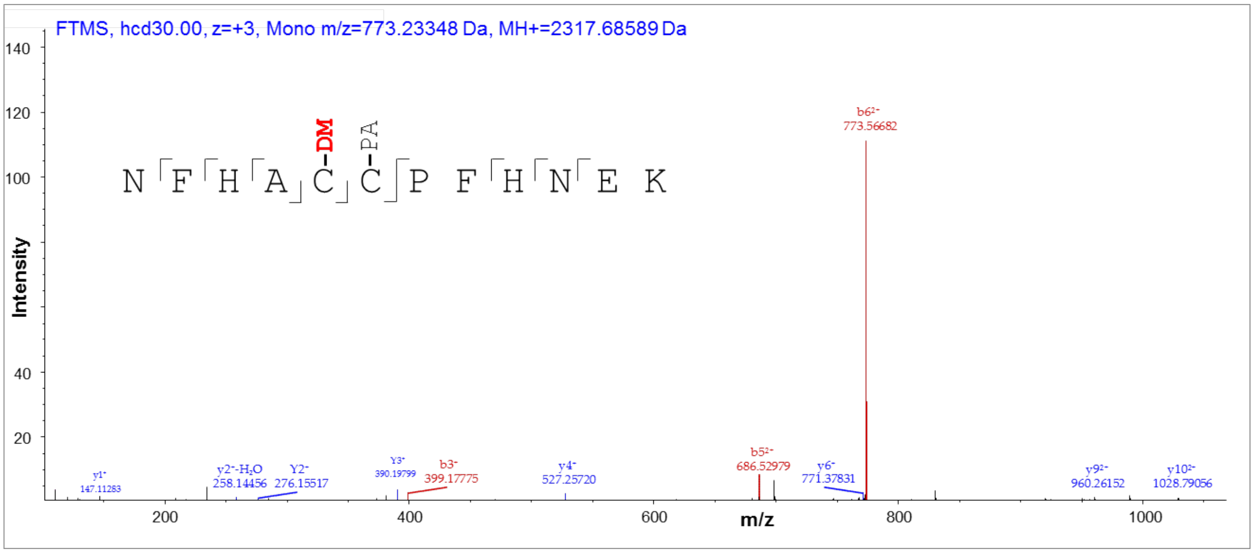

Supplementary Figure S8: MS/MS spectra of DyLight488-modified peptide. DyLight488-labelled primase was digested with trypsin and generated peptides were subjected to LC-MS analysis. Raw data were searched against *E.coli* proteins including a variable modification of 799.74 Da at cysteine residues. MS/MS analyses were done by CID (**a**) or HCD (**b**) fragmentation and y and b ions were indicated in the spectrum by mass, charge and No. Their location in the amino acid sequence of the DyLight488-modified peptide is shown as well as the most likely position of the modifications. DM: DyLight488, PA: propionamidation, DA: deamidation.

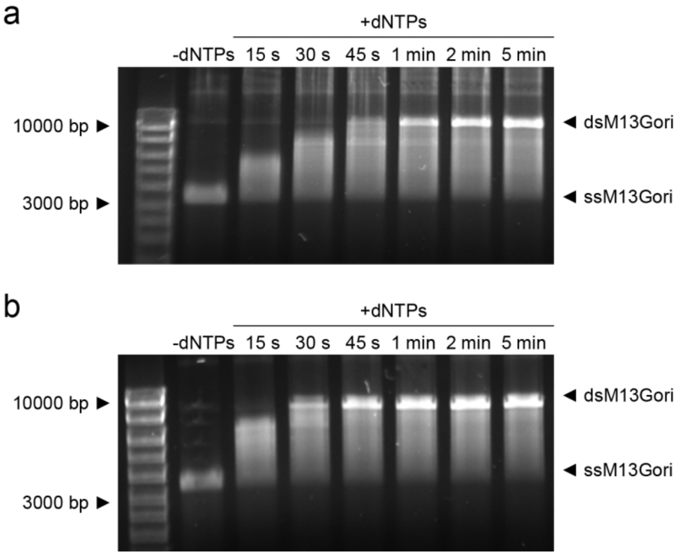

Supplementary Figure S9: DNA replication activity of our prepared proteins using an ssM13Gori template. Details of the experiment are given above. Samples of the replication mixtures were taken at the indicated time points and analysed by agarose gel electrophoresis followed by staining with SYBR Gold. The replication experiment was performed as described below using (**a**) DyLight488-DnaG and (**b**) wild-type DnaG. The molecular weight marker (Hyperladder I, Bioline, UK) provides double-stranded DNA fragments of 200, 400, 600, 800, 1000, 1500, 2000, 2500, 3000, 4000, 5000, 6000, 8000 and 10000 bp.

Replication assays were performed by mixing 5 nM ssM13Gori, 0.8 µM SSB and 10 nM DL-DnaG or DnaG, respectively, in 20 mM Tris/HCl, 150 mM NaCl, 10 mM $MgCl_2$, 1 mM TCEP supplemented with 1 µM BSA and 2 mM ATP and 0.1 mM each of UTP, CTP, GTP. The mixture was incubated for 10 minutes at room temperature to allow for primer synthesis by primase. Then, 10 nM of pol III holoenzyme were added and the DNA replication reaction was started by the addition of 0.1 mM each of dATP, dTTP, dCTP and dGTP. Samples were taken at the indicated time points. The replication reaction was stopped by addition of EDTA to a final concentration of 2 mM to the samples. 10 µl of the samples were mixed with the same volume of a buffer containing 40 mM Tris/HCl, 100 mM EDTA, 0.2 % (w/v) SDS, 50 µg/ml proteinase K, 0.1% (w/v) bromophenol blue, pH 8.0 followed by an incubation for 45 min at 60 °C. 15 µl of the samples were analysed by electrophoresis on a 1% agarose gel in TPE buffer. Following gel electrophoresis, the DNA was stained for 40 min using SYBR Gold (Invitrogen, USA) diluted 1:1000 in TE buffer and the fluorescence was recorded with an excitation wavelength of 312 nm using a Vilbert Lourmat BIOVISION++1000 fluorescence imaging system.

References

1 Zabeau, M. & Stanley, K. K. Enhanced expression of cro-beta-galactosidase fusion proteins under the control of the PR promoter of bacteriophage lambda. *EMBO J.* **1**, 1217-1224 (1982).

2 Chung, C. T., Niemela, S. L. & Miller, R. H. One-step preparation of competent *Escherichia coli*: transformation and storage of bacterial cells in the same solution. *Proc. Natl. Acad. Sci. U S A* **86**, 2172-2175 (1989).

3 Blinkowa, A. L. & Walker, J. R. Programmed ribosomal frameshifting generates the *Escherichia coli* DNA polymerase III gamma subunit from within the τ subunit reading frame. *Nucleic Acids Res.* **18**, 1725-1729 (1990).

4 Tsuchihashi, Z. & Kornberg, A. Translational frameshifting generates the gamma subunit of DNA polymerase III holoenzyme. *Proc. Natl. Acad. Sci. U S A* **87**, 2516-2520 (1990).

5 Scheuermann, R. H. & Echols, H. A separate editing exonuclease for DNA replication: the epsilon subunit of *Escherichia coli* DNA polymerase III holoenzyme. *Proc. Natl. Acad. Sci. U S A* **81**, 7747-7751 (1984).

6 Johanson, K. O., Haynes, T. E. & McHenry, C. S. Chemical characterization and purification of the beta subunit of the DNA polymerase III holoenzyme from an overproducing strain. *J. Biol. Chem.* **261**, 11460-11465 (1986).
